# Comparative NMR-Based Metabolomic and Functional Assessment of Fruit and Vegetable Extracts under Regenerative Agricultural Practices

**DOI:** 10.1021/acs.jafc.6c01741

**Published:** 2026-07-03

**Authors:** Ana Isabel Tristán, Gloria Perazzoli, Ana Cristina Abreu, Ana del Mar Salmerón, Silvia Fernández, Francisco Javier del Águila, Juan Enrique Gázquez, Antonio Fernández, Consolación Melguizo, José Prados, Ignacio Fernández

**Affiliations:** † Department of Chemistry and Physics, Research Centre CIAIMBITAL, University of Almería, Ctra. Sacramento, s/n, 04120 Almería, Spain; ‡ Institute of Biopathology and Regenerative Medicine (IBIMER), Center of Biomedical Research (CIBM), University of Granada, 18100 Granada, Spain; § Instituto de Investigación Biosanitaria de Granada, ibs.GRANADA, 18012 Granada, Spain; ∥ Department of Anatomy and Embryology, Faculty of Medicine, University of Granada, 18071 Granada, Spain; ⊥ Viagro S.A., Ctra. La Cañada-Viator, 04120 Almería, Spain

**Keywords:** Regenerative agriculture, metabolomics, NMR, functional bioassays, health-related properties

## Abstract

Agricultural practices are increasingly shifting toward more sustainable and regenerative approaches; however, their impact on crop metabolism and associated biological effects remains incompletely understood. In this study, ^1^H NMR-based metabolomics combined with functional biological assays was used to investigate metabolic modulation in blueberry, cauliflower, and peach extracts obtained under regenerative and conventional agricultural systems. Metabolic profiling revealed clear, matrix-dependent differences in carbohydrates, amino acids, organic acids, and phenolic-related compounds between cultivation strategies. Multivariate analysis (PLS-DA) showed discrimination between regenerative and conventional samples. Cauliflower exhibited the most pronounced metabolic modulation, followed by peach, whereas blueberry showed more selective changes. Functional assays showed enhanced fibroblast migration in peach extracts, increased cytoprotective and detoxifying responses in cauliflower, and antiangiogenic activity in blueberry samples. Overall, these results suggest that agricultural practices may influence the metabolic and functional properties of plant-derived foods and demonstrate the value of combined metabolomic and biological approaches for the comparative evaluation of regenerative production systems.

## Graphical abstract

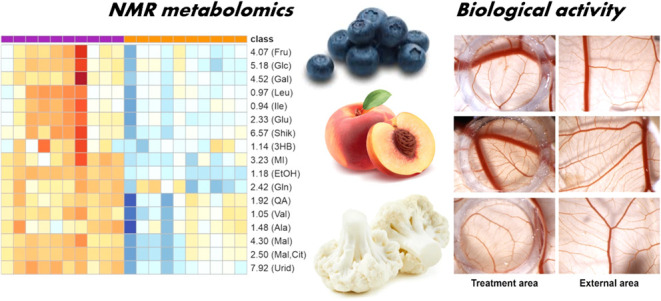

## 1 Introduction

Regenerative agriculture (RA) represents an increasingly adopted agronomic paradigm aimed at restoring soil functionality, enhancing biodiversity, and improving agroecosystem resilience through sustainable management practices. Beyond its environmental benefits, such as carbon sequestration and improved soil structure, RA has also been proposed to influence plant metabolism and, consequently, the nutritional and functional quality of crops.^1^ Growing evidence suggests that regenerative practices can induce measurable metabolic shifts in plants, promoting the accumulation of secondary metabolites such as polyphenols, flavonoids, terpenoids, and phenolic acids, which are widely associated with antioxidant, anti-inflammatory, and cytoprotective activities.

Fruits and vegetables represent highly complex biochemical systems composed of primary metabolites (e.g., sugars, amino acids, organic acids, and lipids) essential for plant growth, together with a diverse array of secondary metabolites that mediate plant–environment interactions. These secondary metabolites contribute to plant defense, signaling, and adaptation to biotic and abiotic stressors, while also playing a central role in the nutritional and functional value of plant-derived foods. Increasing evidence indicates that agricultural practices can modulate both primary and secondary metabolic pathways, thereby shaping the nutritional and health-promoting profiles of edible plant tissues.^2,3^ Because metabolic composition is highly sensitive to environmental inputs, crop management strategies, and cultivar-specific traits, comprehensive metabolomic approaches are required to capture these biochemical changes. In this context, nuclear magnetic resonance (NMR)–based metabolomics offers a robust, quantitative, and highly reproducible platform for the untargeted profiling of complex plant matrices. Its ability to simultaneously detect carbohydrates, amino acids, organic acids, and secondary metabolites without extensive sample manipulation makes it particularly suitable for comparative studies assessing the impact of agronomic practices on fruit and vegetable composition.^3,4^

Although metabolomics has been widely applied to study plant metabolism and agricultural practices, its application to regenerative agriculture systems remains very limited. Most available studies focus on animal-derived products, environmental matrices, or specific agronomic factors rather than plant-based foods under regenerative management.

To the best of our knowledge, only the work by Salmerón et al. has explored the use of metabolomics to investigate plant responses under regenerative agricultural conditions. In that study, ^1^H NMR-based metabolomics was applied to assess metabolic changes in tomato plants under regenerative practices, revealing alterations in primary and secondary metabolism associated with plant health and stress responses.^5^

However, studies integrating metabolomic profiling with functional biological assays to evaluate the impact of regenerative agriculture on plant-derived foods remain limited.

Beyond their nutritional value, plant secondary metabolites have received increasing attention for their potential roles in human health. Polyphenols and flavonoids, for instance, exhibit antioxidant, anti-inflammatory, and cytoprotective activities, and have been implicated in the modulation of key cellular pathways related to oxidative stress, inflammation, and cell proliferation.^6,7^ Compounds such as quercetin, nobiletin, apigenin, luteolin, and fisetin have demonstrated protective effects in various experimental models, including neurodegenerative and oncological contexts.^8,9^ Other flavonoids, including apigenin, luteolin, and fisetin, have demonstrated inhibitory effects on tumor cell migration and invasion by modulating pathways such as TGF-β and uPA/uPAR, and have shown synergistic effects when combined with conventional chemotherapeutic agents.^10–12^ Likewise, phenolic-rich extracts from plant sources such as *Citrullus colocynthis* and *Inula viscosa* have shown antioxidant, anti-inflammatory, and antiproliferative activities, reinforcing the relevance of dietary phytochemicals as contributors to metabolic health.^13,14^ Similarly, polyphenols such as curcumin have been extensively studied for their anticancer properties, including modulation of cell cycle progression and induction of apoptosis in multiple cancer models.^15–17^ Alkaloids such as berberine, together with phenolic acids like ferulic and sinapic acid, have also shown cytotoxic and pro-apoptotic effects in colon cancer cell lines.^18^ Collectively, these findings highlight the importance of understanding how agricultural practices modulate metabolite composition and biological activity.

In this context, the present study investigates how a regenerative agriculture–based cultivation strategy influences the metabolomic profiles and biological activities of three widely consumed plant matrices: blueberry (*Vaccinium corymbosum*), cauliflower (*Brassica oleracea* *var. botrytis*), and peach (*Prunus persica*). Using ^1^H NMR–based metabolomics combined with functional biological assays, we compare extracts obtained under regenerative and conventional cultivation systems. The aim of this study is to determine whether regenerative practices induce reproducible metabolic shifts and whether these changes are associated with measurable effects on antioxidant capacity, enzymatic modulation, cell migration, and angiogenesis-related responses. This combined approach provides a comparative framework to explore potential links between agricultural practices, food quality and related functional properties.

## 2 Materials and Methods

### 2.1 Regenerative Agriculture Conditions

Productive commercial farms of three different crops including peaches (*Prunus persica*), blueberries (*Vaccinium corymbosum*), and cauliflower (*Brassica oleracea* *var. botrytis*) were selected to evaluate the impact of agronomic management on the metabolomic composition and biological activity of the final products (fruits and vegetable). For each crop, two agronomic management systems were compared: (i) a conventional standard management system (control) and (ii) a regenerative agriculture management system certified under the Epigen Healthy Bite (EHB) standard (Viagro S.A., Almería, Spain). To ensure robustness and reproducibility, all field operations were conducted in strict compliance with the guidelines established by the European and Mediterranean Plant Protection Organization (EPPO) (guidelines PP1/152 and PP1/181). The main characteristics of the three crops included in the study are summarized in Table 1. The planting date reported in Table 1 refers to the year in which the regenerative agriculture protocol under the EHB certification was initiated. Accordingly, at the time of harvest considered in this study, blueberry crops had been managed under regenerative agriculture for seven years, whereas cauliflower and peach crops had been under this management system for two and six years, respectively.

**Table 1:** Detail of the Crops Subjected to Regenerative Agriculture Studies

*Crop/Variety*	Location	Soil Texture	Planting date	Harvest date
Blueberry	Moguer, Huelva, Spain	Sandy loam	Sep 2015	14/06/2022
Cauliflower	Las Marismas de Lebrija, Sevilla, Spain	Silty clay	Sep 2021	21/01/2022
Peach	Villanueva de la Serena, Badajoz, Spain	Silt loam	Ene 2016	15–20/07/2022

Each trial consisted of two independent production units, selected based on a high degree of similarity in terms of soil profile, microclimatic conditions, and management history. We used regenerative agriculture (EPI) that employed an alternative set of inputs for crop nutrition and phytosanitary management, all of which were officially certified under the Epigen Healthy Bite standard (EHB-certified). As control treatment (NONEPI) we used the farmer’s standard commercial practices and conventional agricultural inputs. Crop development at each farm was monitored by experienced agricultural engineers with extensive experience in the respective crops, who ensured the use of certified regenerative agriculture inputs as the only significant experimental variable. Meteorological stations were deployed to confirm the comparability of environmental conditions and pest and disease pressure throughout the entire crop cycle at both farms. In addition, both farms received equivalent total nutrient units and were subjected to identical irrigation schedules. Finally, to ensure statistically robust data and avoid the potential within-field variability, a randomized sampling design was applied across the cultivation plots. Thus, representative crop samples were collected from these random blocks, ensuring that the material accurately reflected the entire production unit. All samples were handled following identical preservation and transport protocols and were transferred to the laboratory to maintain their integrity prior to subsequent analyses.

### 2.2 Plant Material and Sample Processing

Peaches, blueberries and cauliflower cultivated under EPI and NONEPI conditions were provided by Viagro S.A. For each crop, individual samples collected from multiple plants across the cultivation plots were selected and combined to obtain representative composite samples for each cultivation condition. Specifically, 40 blueberries, 6 peaches, and 5 cauliflower heads were pooled per group prior to processing. In the case of peaches, fruits were peeled and the stone was removed prior to processing. For cauliflower, four florets were collected from each head to account for within-sample variability. All samples were immediately transported to the laboratory (University of Almería), where they were washed, manually cut into small pieces (except for blueberries), and homogenized by grinding to ensure sample homogeneity. The resulting powders were rapidly stored at −80 °C to minimize metabolic degradation and subsequently lyophilized for 72 h. After freeze-drying, the homogenized material was accurately weighed, aliquoted, and independently extracted for subsequent metabolomic and biological analyses. Each aliquot was processed and analyzed separately by NMR. This experimental design was intended to obtain averaged metabolic profiles representative of each cultivation system while minimizing variability associated with individual samples, sampling, and sample handling. However, this approach does not allow assessment of biological variability between individual fruits or plants.

### 2.3 Sample Preparation for Biological Activity Assays

Freeze-dried powders obtained from each plant matrix (peach, blueberry, and cauliflower) cultivated under EPI and NONEPI conditions were used for the preparation of extracts employed in the biological assays. For each sample, approximately 2 g of lyophilized material were extracted with a water:methanol mixture (1:1, v/v; final volume 40 mL). The extraction was performed under continuous magnetic stirring in a thermostated water bath at 30 °C for 30 min to ensure efficient solubilization of both polar and moderately polar metabolites. Following extraction, the organic solvent was removed under reduced pressure using a rotary evaporator. The remaining aqueous phase was subsequently frozen and lyophilized for 72 h to obtain dry extracts suitable for biological evaluation. All extracts were stored at −20 °C until use. The resulting lyophilized extracts were then shipped to the collaborating laboratory at the University of Granada, where a series of biological activity assays were conducted. These included *in vitro* wound-healing assays, cell-based antioxidant activity measurements, evaluation of detoxifying enzyme induction, cytosolic fraction isolation, assessment of NAD­(P)­H:quinone oxidoreductase 1 (NQO1) activity, glutathione S-transferase (GST) activity, and *in vivo* angiogenesis evaluation using the chick chorioallantoic membrane (CAM) model.

### 2.4 Sample Preparation for NMR-Based Metabolomic Analysis

All metabolomic analyses were performed at the University of Almería. Sample preparation for NMR-based metabolomic analysis was carried out following established and validated protocols for plant-derived matrices, with minor adaptations to the specific characteristics of each sample type.^5,19–21^ Lyophilized powders obtained from peach, blueberry, and cauliflower samples were used for extraction, employing 35 mg for blueberry and cauliflower and 25 mg for peach, to account for differences in matrix density and extractability. Each sample was extracted using 0.8 mL of a 1:1 (v/v) mixture of deuterated methanol (CD_3_OD) and phosphate buffer prepared in D_2_O (pH 6.0). The buffer contained 0.1% (w/v) TSP-*d*_4_ as an internal chemical shift reference and quantitative standard, as well as 90 μM sodium azide (NaN_3_) to inhibit enzymatic activity during sample handling. The mixtures were sonicated for 20 min to enhance metabolite extraction, followed by vortex agitation at 600 rpm for 10 min to ensure homogenization. Subsequently, the extracts were centrifuged at 13,500 rpm for 5 min at room temperature to remove insoluble material. An aliquot of 500 μL of the clear supernatant was then transferred into 5 mm NMR tubes for spectral acquisition. All samples were prepared under identical conditions to ensure analytical reproducibility and allow reliable comparative metabolomic analysis between agricultural treatments. In addition, independent extractions of homogenized samples were performed to assess analytical reproducibility, yielding consistent spectral profiles across replicates.

### 2.5 NMR Experiments

All ^1^H NMR spectra were acquired on a Bruker AVANCE III 600 MHz spectrometer equipped with a 5 mm QCI cryoprobe and a temperature-controlled SampleJet autosampler. Spectra were recorded at 300 ± 0.1 K under static conditions. Water suppression was achieved using a standard presaturation pulse sequence (noesygppr1d), with selective irradiation of the residual H_2_O signal during both relaxation and mixing periods. Each spectrum was acquired using 32 scans and 4 dummy scans, over a spectral width of 20 ppm with 64 K data points. The acquisition time was 2.73 s and the relaxation delay was set to 5.0 s, resulting in a digital resolution of 0.36 Hz. The spectrometer was locked on CD_3_OD. Spectral processing included automatic phase and baseline correction, followed by manual inspection to ensure spectral quality and consistency across all samples. Given the comparative nature of the study, metabolite levels are reported as relative signal intensities rather than absolute concentrations. Metabolite identification was supported by two-dimensional NMR experiments, including ^1^H–^1^H TOCSY, ^1^H–^13^C HSQC, and ^1^H–^13^C HMBC, using standard Bruker pulse sequences. Assignments were confirmed through comparison with reference spectra from the Chenomx NMR Suite (Edmonton, Canada), the Human Metabolome Database (HMDB), and previously published studies.^22,23^

### 2.6 Cell Culture

All biological assays were carried out at the University of Granada using the aqueous–methanolic extracts prepared as described in Section 2.3. L929 normal mouse fibroblast and T84 and HT-29 human colonic adenocarcinoma cell lines were used for the experiences. All cell lines were provided by the American Type Culture Collection (ATCC, USA). Dulbecco’s Modified Eagle’s Medium (DMEM) (Sigma-Aldrich, Madrid, Spain) was established for maintaining cells. It was supplemented with 10% fetal bovine serum (FBS) and 1% penicillin-streptomycin (Sigma–Aldrich, Madrid, Spain). The culture atmosphere was established in 37 °C and 5% CO_2_.

### 2.7 Cell Viability Assay

T84 and HT-29 cells were seeded in 48-well plates at a density of 5 × 10^3^ and 1.5 × 10^4^ cells/well, respectively. 24 h after seeding, the cells were exposed to concentrations between 100 and 1000 μg/mL of each extract over a total of 72 h. The cell proliferation percentages were determined using a modified colorimetric assay involving sulforhodamine B (SRB) (Sigma-Aldrich, Madrid, Spain). To fix the cells, a 10% trichloroacetic acid (TCA) solution (Sigma-Aldrich, Madrid, Spain) was applied as a cell fixative for 20 min at 4 °C. Subsequently, a staining solution containing 0.4% SRB diluted in 1% acetic acid was used to stain the cells for 20 min at room temperature with agitation. Trizma (10 mM, pH 10.5) (Sigma-Aldrich, Madrid, Spain) was then utilized to solubilize the SRB, and the optical density (OD) was measured at 492 nm using a spectrophotometer, specifically the EX-Thermo Multiskan (Waltham, Massachusetts, USA).

### 2.8 Wound-Healing Assay

L929 and T84 cells were seeded in 6-well plates at a density of 3 × 10^5^ cells/well for a full monolayer in 1 mL of complemented DMEM. The next day, a wound was made through the central part of each well following the methodology established by Grada et al.^24^ After washing with PBS, 1 mL of DMEM without FBS was added along with a treatment of each extract at nontoxic doses (10 μg/mL). During the following 72h, the cell migration progress was monitored each 24h through images captured with a DM IL LED microscope (Leica, Wetzlar, Germany). The images were analyzed using a specific ImageJ plugin (NIH, USA) to calculate the percentage of cell migration by measuring the cell-free area^25^

### 2.9 Cell-Based Antioxidant Analysis

HT29 cells were seeded in 96-well plates at a density of 2.5 × 10^4^ cells/well in 150 μL of DMEM supplemented with appropriate additives. After 24 h, the culture medium was replaced with serum-free DMEM. On the following day, treatments of each extract (EPI-extract and NONEPI-extract) were applied using two nontoxic doses for a duration of 24 h (5 and 10 μg/mL). Following this treatment, the medium containing the treatments was discarded, and fresh serum-free medium was added. Some wells were also treated with varying concentrations of H_2_O_2_ (1.2 mM). After 6 h, the medium was once again replaced with fresh serum-free medium and incubated for an additional 12 h. To assess cell viability, the MTT (3-(4,5-Dimethylthiazol-2-yl)-2,5-Diphenyltetrazolium Bromide) protocol was employed. Briefly, 30 μL of MTT solution was added to each well and incubated under culture conditions for 2.5 h. Subsequently, the medium was discarded, and a mixture of 200 μL of dimethyl sulfoxide (DMSO) and 25 μL of Sorensen’s glycine buffer (containing glycine 0.1 M, NaCl 0.1 M, pH 10.5 with 0.1 NaOH) was added to dissolve the formazan crystals. The OD of the wells was measured at 570 nm, with a reference wavelength of 690 nm, using the EX-Thermo Multiskan spectrophotometer (Waltham, Massachusetts, USA).

### 2.10 Capacity for Inducing Detoxifying Enzymes Analysis

#### 2.10.1 Cytosolic Fraction Extraction

The cellular cytosolic content was extracted following the methodology previously developed by our group.^26^ The HT29 cell line was seeded in 6-well plates at a density of 5 × 10^5^ cells/well in 1.5 mL of culture medium in duplicate and incubated for 24 h. Then, cells were exposed to noncytotoxic concentrations (5 μg/mL) of each extract and DL-Sulforaphane (SFN) (Sigma-Aldrich, Madrid, Spain) 5 μM which was used as positive control. After 48 h of EPI-extract and NONEPI-extract treatment, the cells were washed, trypsinised and centrifuged. After being washed twice with PBS, the cell pellet was resuspended in 500 μL Tris-HCl buffer (25 mM, pH 7.4) and sonicated in ice for 20 s. Finally, after centrifuging cells during 5 min at 10,000*g* and 4 °C, supernatant from the cytosol was preserved at −80 °C. Protein concentration was assessed utilizing Bradford Reagent (Bio-Rad, California, EEUU).

#### 2.10.2 NAD­(P)­H: Quinone Oxidoreductase Test

The NAD­(P)­H: quinone oxidoreductase (QR) activity was measured colorimetrically because of the reduction of 2.6-dichloroindophenol (DCIP) (molar extinction 0.0205 μmol^–1^/cm^–1^) (Sigma-Aldrich, Madrid, Spain) carried out by the QR, which results in a decrease of the optical density. To conduct the experiment, a reaction mixture was prepared by combining 881.5 μL of 25 mM Tris-HCl solution (pH 7.4), 60 μL of 1 mg/mL Bovine Serum Albumin (BSA) (Sigma-Aldrich, Madrid, Spain), 2.5 μL of Tween (20%) (Bio-Rad, California, EEUU), 5 μL of 1 mM Flavin adenine dinucleotide disodium (FAD) (Sigma-Aldrich, Madrid, Spain), 10 μL of 20 mM β-nicotinamide adenine dinucleotide (NADH) (Sigma-Aldrich, Madrid, Spain), and 16 μL of 5 mM DCIP. Subsequently, 25 μL of cytosolic fraction sample was introduced to a plastic cuvette containing 975 μL of the reaction mixture, and the absorbance at 600 nm was recorded every minute for a duration of 5 min in a UV–vis Spectrophotometer UV-1900i (Shimadzu, Duisburg, Germany). The QR activity was determined by calculating the decrease in absorbance per minute per milligram of total protein and then comparing cells exposed to the EPI-extract, NONEPI-extract and untreated cells.

#### 2.10.3 Glutathione S-Transferase Assay

The activity of the glutathione S-transferase (GST) enzyme was assessed by measuring the colorimetric increase resulting from the GST catalyzed reaction between reduced glutathione (GSH) (Sigma-Aldrich, Madrid, Spain) and the GST substrate, 1-chloro-2,4- dinitrobenzene (CDNB) (molar extinction 0.0096 μmol^–1^/cm^–1^) (Sigma-Aldrich, Madrid, Spain). The reaction mixture, consisting of 870 μL of 100 mM phosphate buffer (pH 6.5), 20 μL of 50 mM CDNB and 10 μL of 100 mM GSH, was incubated at 30 °C for 5 min in a UV–vis Spectrophotometer UV-1900i. Finally, 100 μL of cytosolic fraction sample was introduced to a quartz cuvette containing 900 μL of the reaction mixture and the absorbance at 340 nm was recorded every minute for a duration of 5 min using a UV–vis Spectrophotometer UV-1900i. The GST activity was determined by calculating the increase in absorbance per minute per milligram of total protein and then comparing EPI-extract and NONEPI-extract treated cells with untreated cells.

### 2.11 Chick Chorioallantoic Membrane (CAM) Assay for Angiogenesis Test

Fertilized eggs were procured from a certified poultry farm. As shown in Figure 1, the experimental timeline commenced on day 0, during which the eggs were subjected to a cleaning procedure using 70% ethanol. Subsequently, they were placed in an incubator set at a controlled temperature of 37.0 °C and appropriate humidity levels. For the next 3 days the eggs were turned 180° thrice a day. On day 3, 2 mL of albumen was carefully extracted from the apex of each egg and a window measuring 1.5 cm^2^ was strategically created on the side of the eggshell and a polypropylene ring was placed over the CAM. To protect the window, it was securely covered with tape before returning the eggs to the incubator, ensuring they were placed in a horizontal position to continue their incubation process. On day 7, randomly assigned viable eggs into 7 groups (*n* = 7 for Peach, Blueberries and Cauliflower NONEPI-extract; *n* = 8 for Peach, Blueberries and Cauliflower EPI-extract, and *n* = 5 for control (untreated) eggs) and treated into de ring with 100 μg/mL of each extract in a volume of 40 μL while the negative control was also treated with the same volume of PBS. Following a 72 h incubation period, the CAM region of available eggs (*n* = 7 for Peach NONEPI-extract and Cauliflower EPI-extract; *n* = 5 for Blueberries NONEPI-extract; *n* = 4 for Cauliflower NONEPI-extract and control (untreated) eggs; *n* = 6 for Peach EPI-extract and *n* = 8 for Blueberries EPI-extract) was photographed in and outside the ring against a white background using a Motic SMZ-171 stereo microscope (Motic, Barcelona, Spain). To analyze the obtained images, the “Vessel Analysis” plugin from FIJI was employed.

**Figure 1:**
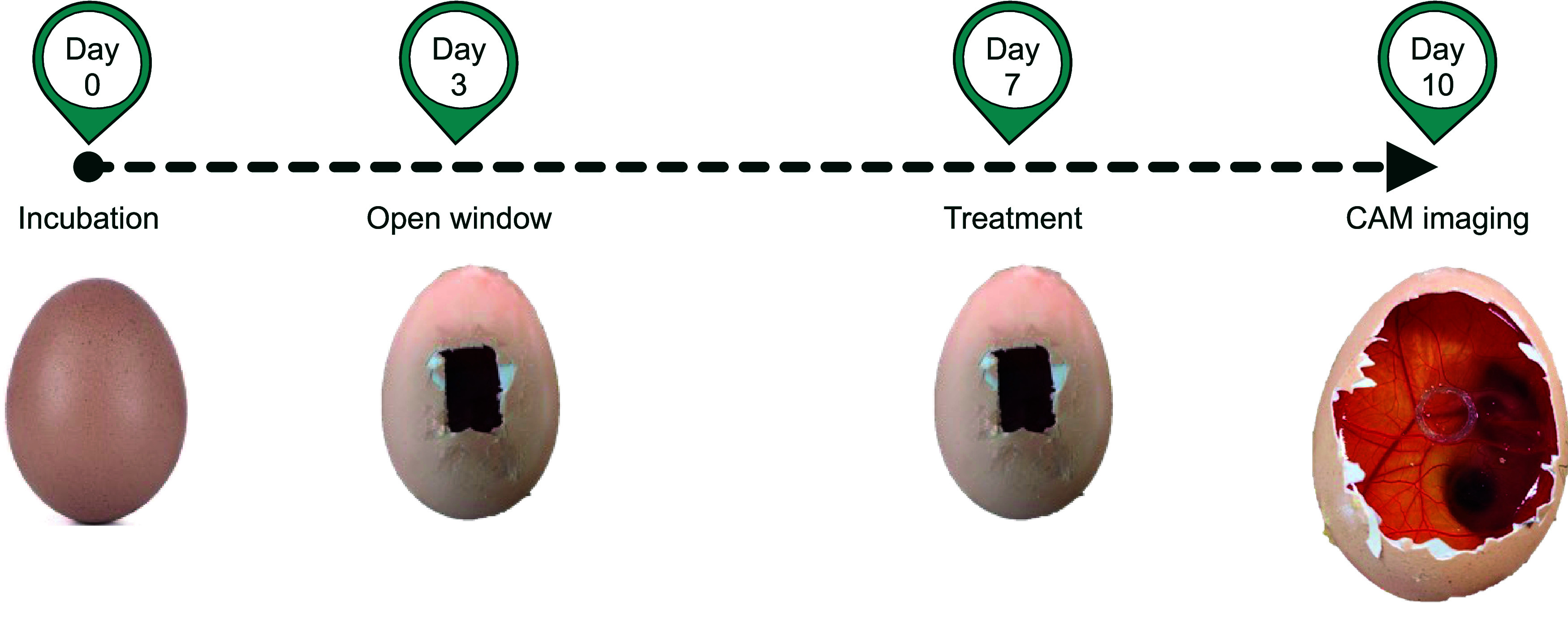
Methodology used for the angiogenesis assay in CAM.

### 2.12 Statistical Analysis

Raw ^1^H NMR spectra were processed and bucketed using AMIX software (version 3.9.15, Bruker). Variable-size bucketing was applied based on metabolite signal regions, excluding the residual solvent (methanol) and water suppression regions. Bucket integrals were normalized to the TSP signal prior to statistical analysis. Statistical analysis of the NMR metabolomics data was performed using SIMCA (version 18, Sartorius). Data structure and analytical reproducibility were initially evaluated by principal component analysis (PCA). Supervised multivariate analysis was performed using partial least-squares discriminant analysis (PLS-DA), constructed independently for each fruit or vegetable matrix to assess metabolic differences between NONEPI and EPI samples. Scaling was performed using unit variance (UV) to give equal weight to all variables, and model performance was evaluated using 7-fold cross-validation, reporting *R* ^2^ (explained variance) and *Q* ^2^ (predictive ability). Model robustness and the risk of overfitting were further assessed using permutation tests (*n* = 200) and CV-ANOVA. In permutation testing, models built with randomly permuted class labels showed lower *Q* ^2^ values compared to the original model, and *Q* ^2^ intercepts below zero were considered indicative of model validity. Discriminant metabolites (variables with a variable importance in projection, VIP > 1) were subsequently represented using contribution plots derived from the validated PLS-DA models. Heatmaps were generated in MetaboAnalyst 6.0 to visualize relative NMR signal intensities of discriminant metabolites. Data were autoscaled prior to visualization, and hierarchical clustering was not applied. Only NMR buckets showing statistically significant differences between groups according to Student’s *t*-test, with false discovery rate (FDR) correction (*p* < 0.05), were included.

Statistical analysis of the biological assays was performed using the Statistical Package for the Social Sciences (SPSS) software (version 26.0). Results are expressed as the mean ± standard deviation (SD). Differences between EPI and NONEPI samples were evaluated using Student’s *t*-tests, with statistical significance set at α = 0.05.

### 2.13 Pathway Analysis

Pathway analysis was performed using the Metabolomics Pathway Analysis (MetPA) module implemented in MetaboAnalyst (https://www.metaboanalyst.ca/), following a previously described approach.^21^ Identified metabolites showing significant differences between EPI and NONEPI samples were used as input.

Pathways were mapped using the *Arabidopsis thaliana* library as a reference model, as no specific fruit-related pathway library is available. Pathway analysis was performed using a hypergeometric test for over-representation analysis combined with pathway topology analysis.

Pathway analysis results were evaluated based on *p*-values, Holm-adjusted *p*-values, false discovery rate (FDR), and pathway impact values.

## 3 Results

### 3.1 NMR Spectral Profiles and Metabolite Identification of Peach, Blueberry, and Cauliflower

Representative ^1^H NMR spectra of peach, blueberry and cauliflower extracts revealed distinct metabolic signatures characteristic of each matrix, as it is represented in Figure 2. All spectra showed signals corresponding to major classes of primary metabolites, including organic acids (e.g., citric, malic, succinic, acetic, 3-hydroxybutyric, formic, fumaric, quinic and shikimic acids), amino acids (e.g., alanine, valine, leucine, isoleucine, threonine, glutamate, glutamine, GABA, arginine, tyrosine, phenylalanine, tryptophan, lysine, histidine, proline, aspartate, asparagine), and sugars, such as glucose, fructose and sucrose. In addition, aromatic signals associated with phenolic compounds were observed predominantly in blueberry extracts (e.g., flavonoid-type phenolic compounds), in agreement with the well-known phenolic-rich profile of berry fruits.^23,27^

**Figure 2:**
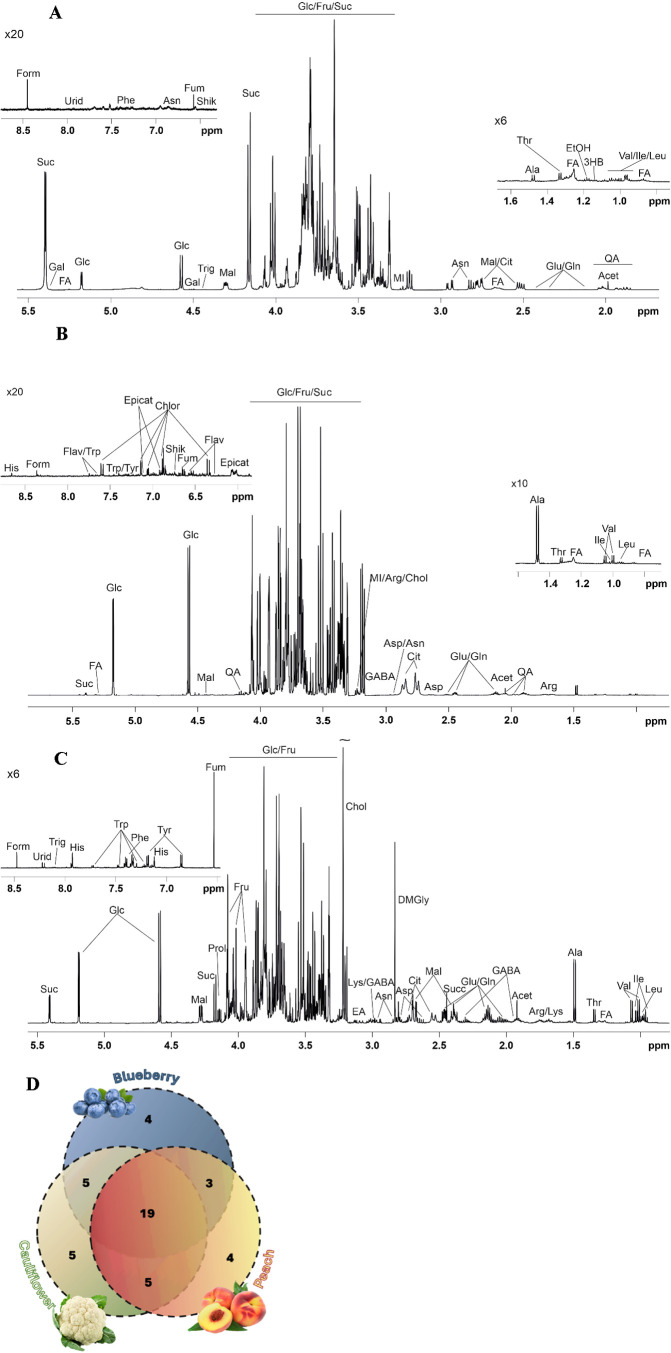
Representative ^1^H NMR spectra of (A) peach, (B) blueberry and (C) cauliflower extracts. Major metabolite resonances were assigned and labeled based on characteristic chemical shifts and supported by 2D NMR experiments and reference databases. Full spectra are shown together with expanded sections of the aromatic region to facilitate visualization. (D) Venn diagram of the distribution of metabolites identified by ^1^H NMR across the three matrices analyzed: peach, blueberry and cauliflower. The diagram highlights metabolites unique to each fruit or vegetable as well as those shared between two or all three matrices, reflecting both common primary metabolic features and matrix-specific chemical signatures.

A total of 30 metabolites were identified in peach, 31 in blueberry and 34 in cauliflower extracts, based on chemical shift patterns, multiplicities and 2D NMR experiments, and supported by spectral databases. While most primary metabolites were common to all three matrices, as shown in the Venn diagram of Figure 2, each sample type exhibited characteristic features: peach spectra were dominated by carbohydrates resonances, blueberry extracts displayed some aromatic and phenolic signals, and cauliflower extracts showed higher levels of amino and organic acids. The spectral information on the three matrices is detailed in Table S1. The bucketed ^1^H NMR spectra obtained from all samples were then used to perform multivariate analyses to evaluate treatment-related metabolic differences.

### 3.2 Multivariate Analysis of Agricultural Treatment

Multivariate analysis was conducted to explore the metabolic differences between conventional (NONEPI) and regenerative (EPI) samples within each fruit or vegetable matrix. Principal component analysis (PCA) was first performed separately for peach, blueberry and cauliflower to explore the internal structure of each data set (Figure S1). To assess the effect of the agricultural treatment, partial least-squares discriminant analysis (PLS-DA) models were then constructed independently for each matrix (Figure 3). For all three fruits and vegetables, the PLS-DA score plots showed a clear separation between EPI and NONEPI samples, indicating treatment-associated metabolic differences. Model performance metrics (*R* ^2^ and *Q* ^2^), obtained through 7-fold cross-validation, together with permutation tests (*n* = 200, Figure S2), supported the validity of the models. In all cases, permuted models yielded lower *Q* ^2^ values than the original model, and *Q* ^2^ intercepts were below zero, suggesting that the observed class separation was not due to random chance (see values in Figure 4). Similar separation trends were also observed in the corresponding PCA models (Figure S1), supporting that the discrimination is not solely driven by supervised analysis. These results demonstrate that the metabolic profiles of peach, blueberry and cauliflower are consistently modulated by the agricultural treatment. The specific metabolites driving these differences are described in the following section.

**Figure 3:**
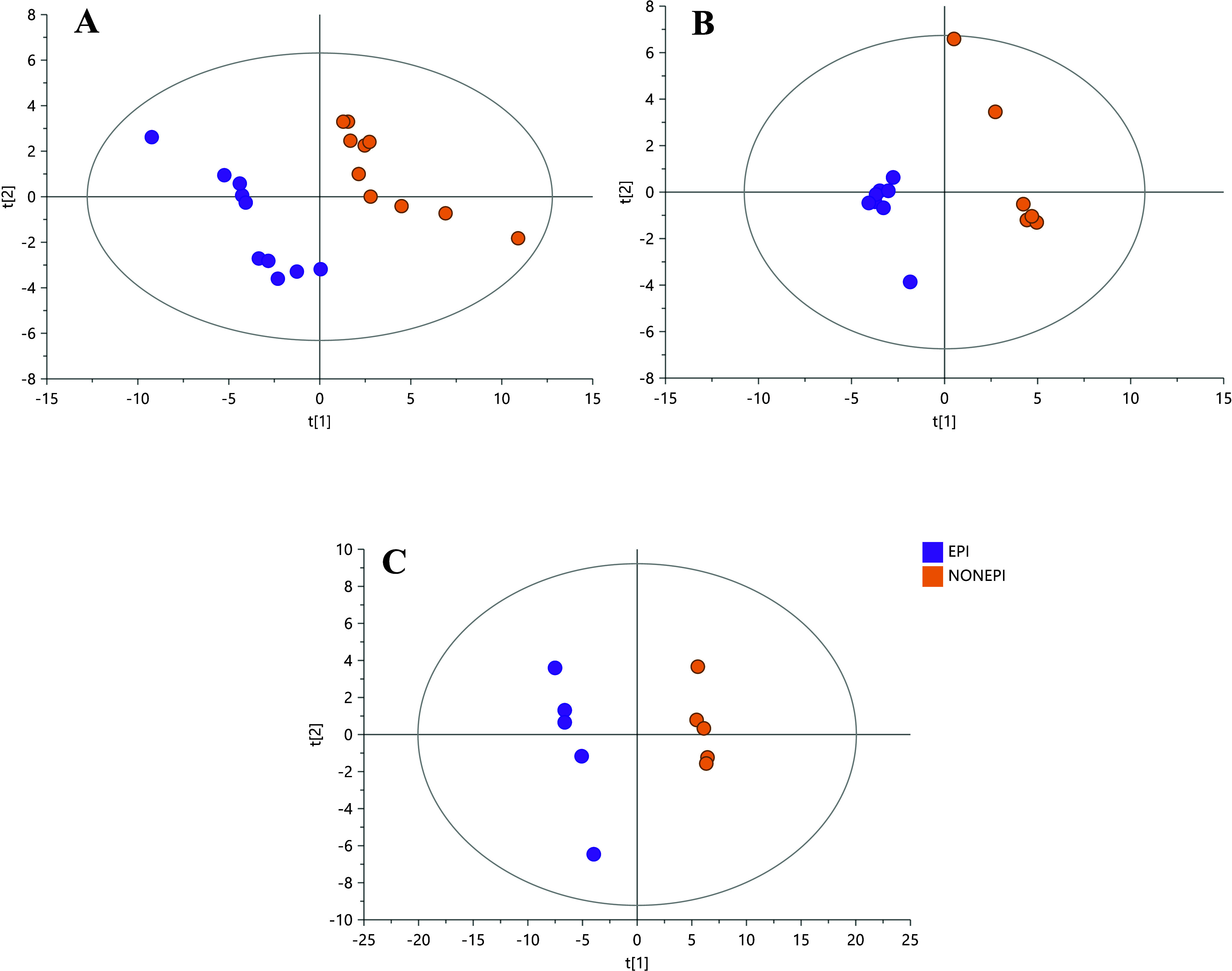
PLS-DA scores plots based on bucketed ^1^H NMR data for (A) peach (*R* ^2^ *X* = 0.80, *R* ^2^ *Y* = 0.99, *Q* ^2^(cum) = 0.95, *p* (CV-ANOVA) < 0.001), (B) blueberry (*R* ^2^ *X* = 0.59, *R* ^2^ *Y* = 0.98, *Q* ^2^(cum) = 0.95, *p* (CV-ANOVA) < 0.001) and (C) cauliflower (*R* ^2^ *X* = 0.76, *R* ^2^ *Y* = > 0.99, *Q* ^2^(cum) = 0.98, *p* (CV-ANOVA) < 0.001). Discrimination between EPI and NONEPI samples was validated by permutation testing.

**Figure 4:**
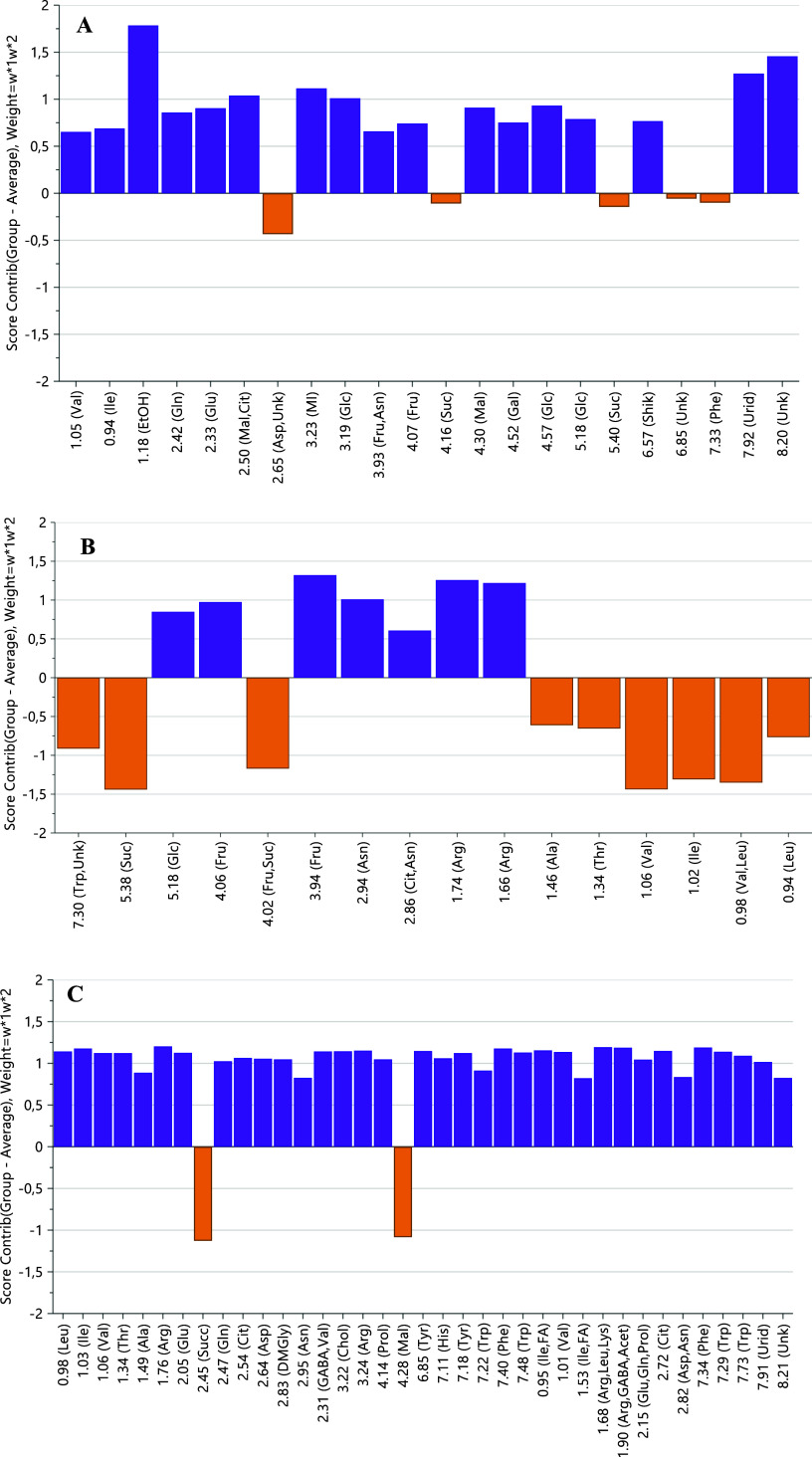
Contribution plots derived from the validated PLS-DA models for (A) peach, (B) blueberry and (C) cauliflower. The plots display the metabolite signals contributing most strongly to class separation between EPI and NONEPI samples. Positive (colored in purple) and negative (colored in orange) contributions indicate metabolites associated with EPI and NONEPI samples, respectively. Only metabolites with VIP scores >1.0 are shown.

### 3.3 Discriminant Metabolites and Treatment-Associated Metabolic Patterns

The metabolites contributing most strongly to the discrimination between EPI and NONEPI samples were identified using contribution plots (VIP > 1) derived from the validated PLS-DA models for each matrix (Figure 4A–C). For peach, blueberry and cauliflower, several metabolites were found to drive treatment-related separation, although both the magnitude and direction of their contributions differed among the three matrices. To visualize these metabolic changes at a global level, heatmaps were generated for each matrix (Figure 5A–C), revealing clear treatment-dependent patterns characterized by coordinated increases or decreases of specific metabolite groups.

**Figure 5:**
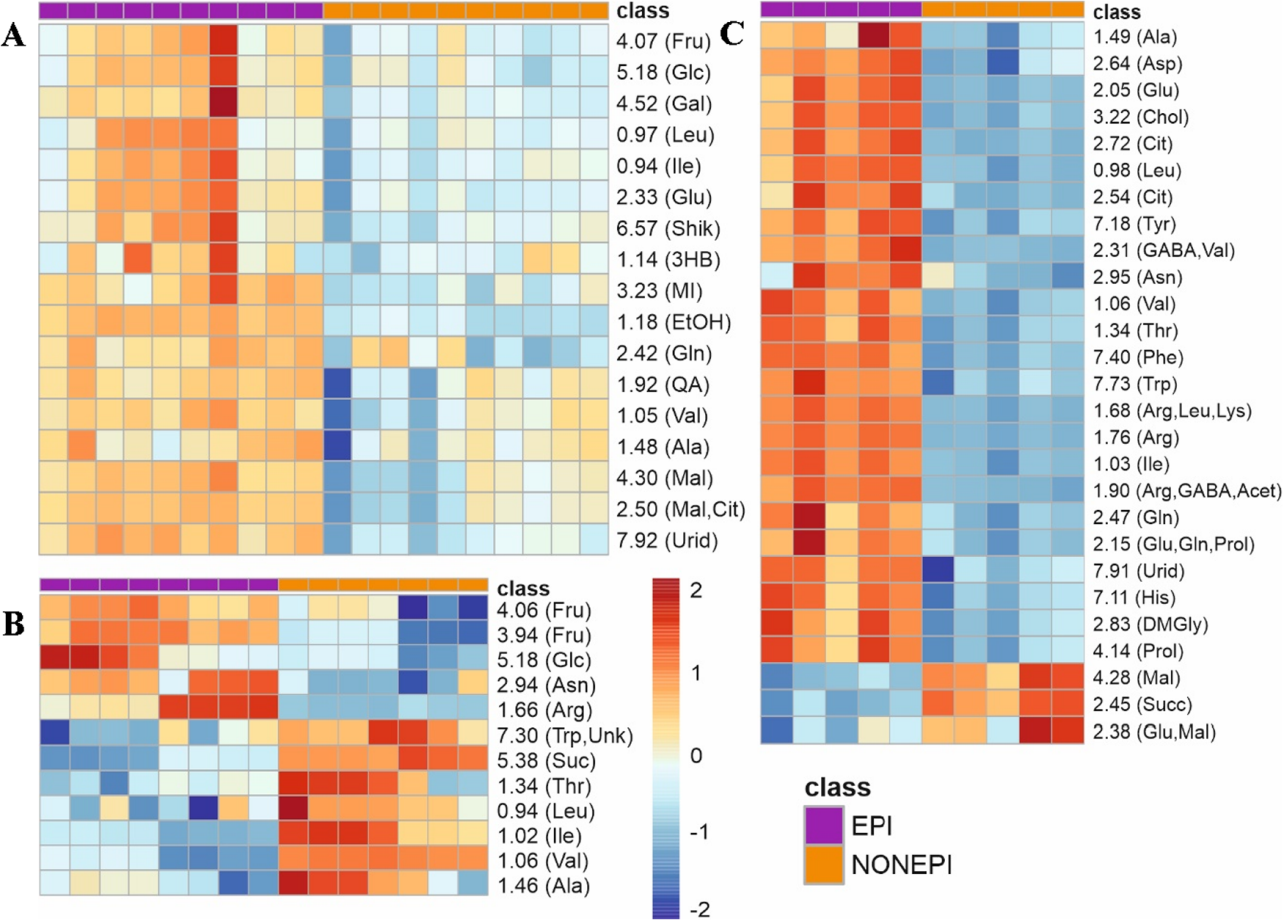
Heatmaps showing the relative levels of discriminant metabolites in (A) peach, (B) blueberry, and (C) cauliflower samples, grown under EPI and NONEPI agricultural practices. Only metabolites showing statistically significant differences between EPI and NONEPI samples according to univariate analysis (*t* test/, *p* (FDR) < 0.05) are displayed. Data were normalized and scaled as described in the Section 2, and color intensity represents relative abundance of metabolite signals across samples.

In blueberry extracts, treatment-associated metabolic differences were primarily driven by changes in sugars and amino acids. Specifically, EPI samples showed higher relative levels of glucose, fructose, asparagine and arginine, whereas NONEPI samples displayed increased levels of sucrose, threonine, alanine, valine, isoleucine and leucine. These results indicate a mixed pattern of carbohydrate- and nitrogen-related metabolites discriminating between treatments (Figures 4B and 5B). Pathway analysis for blueberry indicated that the most affected pathways were related to carbohydrate metabolism (Figure S3 and Table S2). In particular, starch and sucrose metabolism and galactose metabolism were identified as the main altered pathways. In contrast to the more selective response observed in blueberry, cauliflower exhibited the most extensive treatment-related modulation. EPI samples showed higher levels of multiple amino acids (leucine, isoleucine, alanine, valine, threonine, arginine, glutamate, glutamine, aspartate, asparagine, proline, tyrosine, histidine, tryptophan and phenylalanine), citrate, dimethylglycine, choline and uridine. Conversely, NONEPI samples were characterized by increased levels of the two organic acids malate and succinate. These results indicate that treatment-specific separation in cauliflower was dominated by variation in nitrogen-containing metabolites and TCA-related intermediates (Figures 4C and 5C). Pathway analysis of cauliflower revealed a broader metabolic impact compared to the other matrices, predominantly involving amino acid metabolism and central carbon metabolism (Figure S3 and Table S2). The most affected pathway was alanine, aspartate and glutamate metabolism, showing both high enrichment and pathway impact. In addition, pathways related to glyoxylate and dicarboxylate metabolism, arginine biosynthesis, and the TCA cycle were also identified. Peach extracts showed an intermediate pattern of metabolic separation between those seen in blueberry and cauliflower. EPI samples exhibited higher levels of some amino acids (valine, isoleucine, glutamate and glutamine), sugars (glucose, fructose and galactose), ethanol, shikimate, malate, myo-inositol, uridine and one unidentified aromatic resonance, tentatively assigned to a nucleoside/nucleotide–related compound or nitrogenous base. In contrast, NONEPI samples showed higher levels of aspartate, sucrose and phenylalanine. These differences were mainly associated with sugars, amino acids and organic acids, demonstrating a mixed directionality between treatments (Figures 4A and 5A). In agreement with the metabolic differences described above, pathway analysis for peach indicated that the most affected pathways were related to carbohydrate metabolism and amino acid metabolism (Figure S3 and Table S2). In particular, galactose metabolism showed the strongest enrichment, followed by starch and sucrose metabolism, and pathways associated with amino acid metabolism, including arginine biosynthesis and alanine, aspartate and glutamate metabolism. In general, sugars and amino acids were the most consistently modulated metabolite classes. However, the direction and magnitude of change varied by fruit or vegetable type. Taken together, these results indicate distinct metabolic responses to agricultural treatment across matrices.

### 3.4 Analysis of Antiproliferative Activity

Two different human colorectal cancer cell lines were selected to evaluate the antiproliferative capacity of peach, blueberries and cauliflower from both EPI and NONEPI-extracts, therefore, doses up to 1000 μg/mL of each extract were tested. The results obtained showed that none of the natural extracts tested presented cytotoxic activity in any of the two cell lines tested as shown in Figure 6.

**Figure 6:**
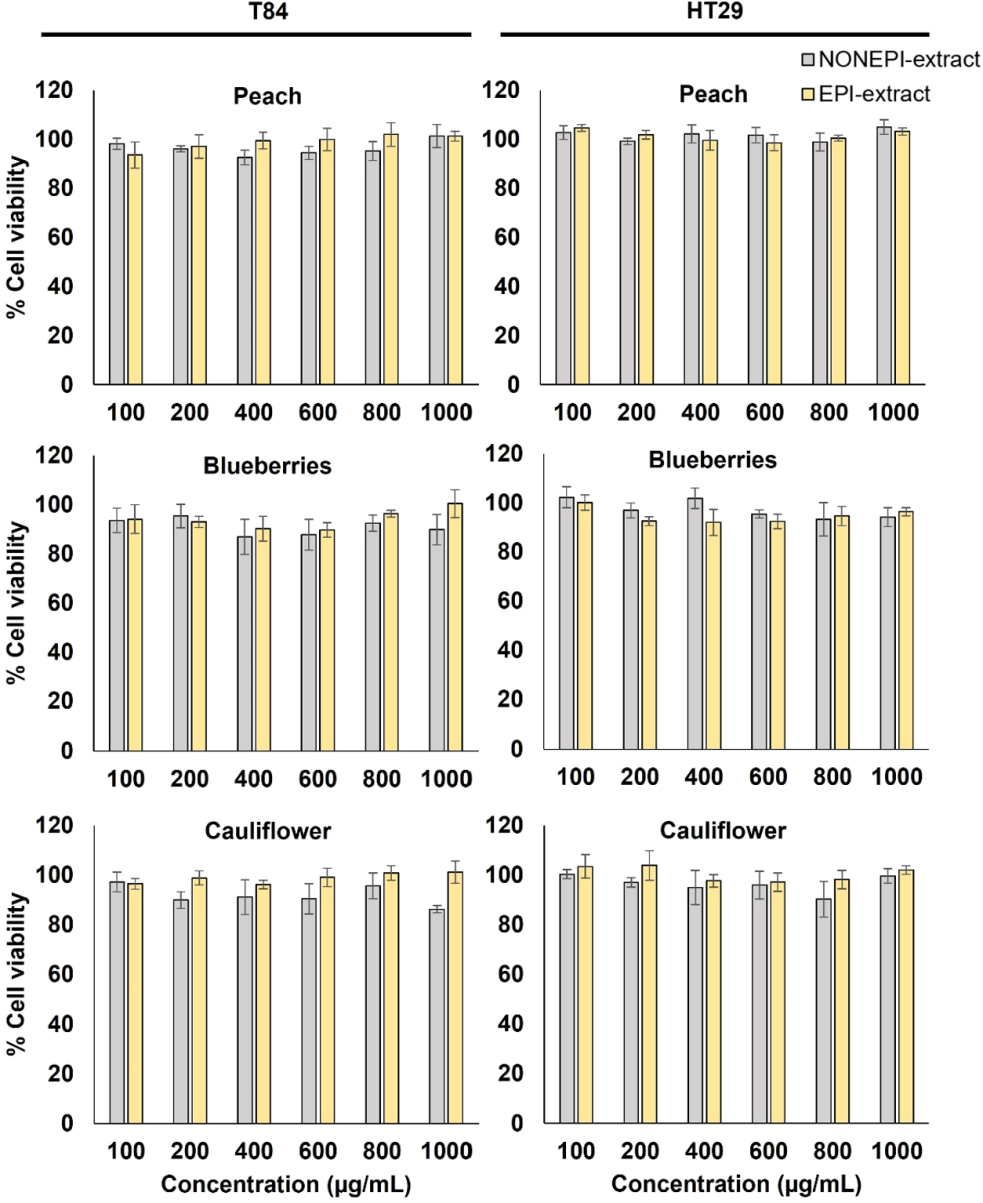
Effect of natural extracts on cell proliferation. An increasing range of concentrations were tested in the colon cell lines T84 and HT29. Data are presented as the mean ± standard deviation of triplicate cultures.

### 3.5 Modulation of the Cell Migratory Capacity by the Extracts

To assess the potential effects of the obtained extracts on cell migration, a wound healing assay was conducted using a normal fibroblast cell line (L929) and a human colon carcinoma cell line (T84). As shown in Figure 7, the most notable effect was observed with the peach-EPI-extract, which significantly enhanced cell migration, particularly after 48 h of treatment (58.8%) compared to the NONEPI-extract (44.8%) (*p* < 0.05). This stimulatory effect persisted at 72 h and was 8.1% greater than that induced by the peach- NONEPI-extract (*p* < 0.05). The blueberry- NONEPI-extract slightly modulated L929 cell migration, whereas both cauliflower extracts (EPI and NONEPI) produced no significant differences compared with the control. Conversely, when T84 cells were analyzed, only the blueberry extracts affected cell migration, with the NONEPI-extract being the most effective, reducing migration by up to 25.8% after 48 h of treatment relative to the control (*p* < 0.01). Neither peach nor cauliflower extracts, in either the EPI or NONEPI form, exhibited significant effects on T84 cell migration.

**Figure 7:**
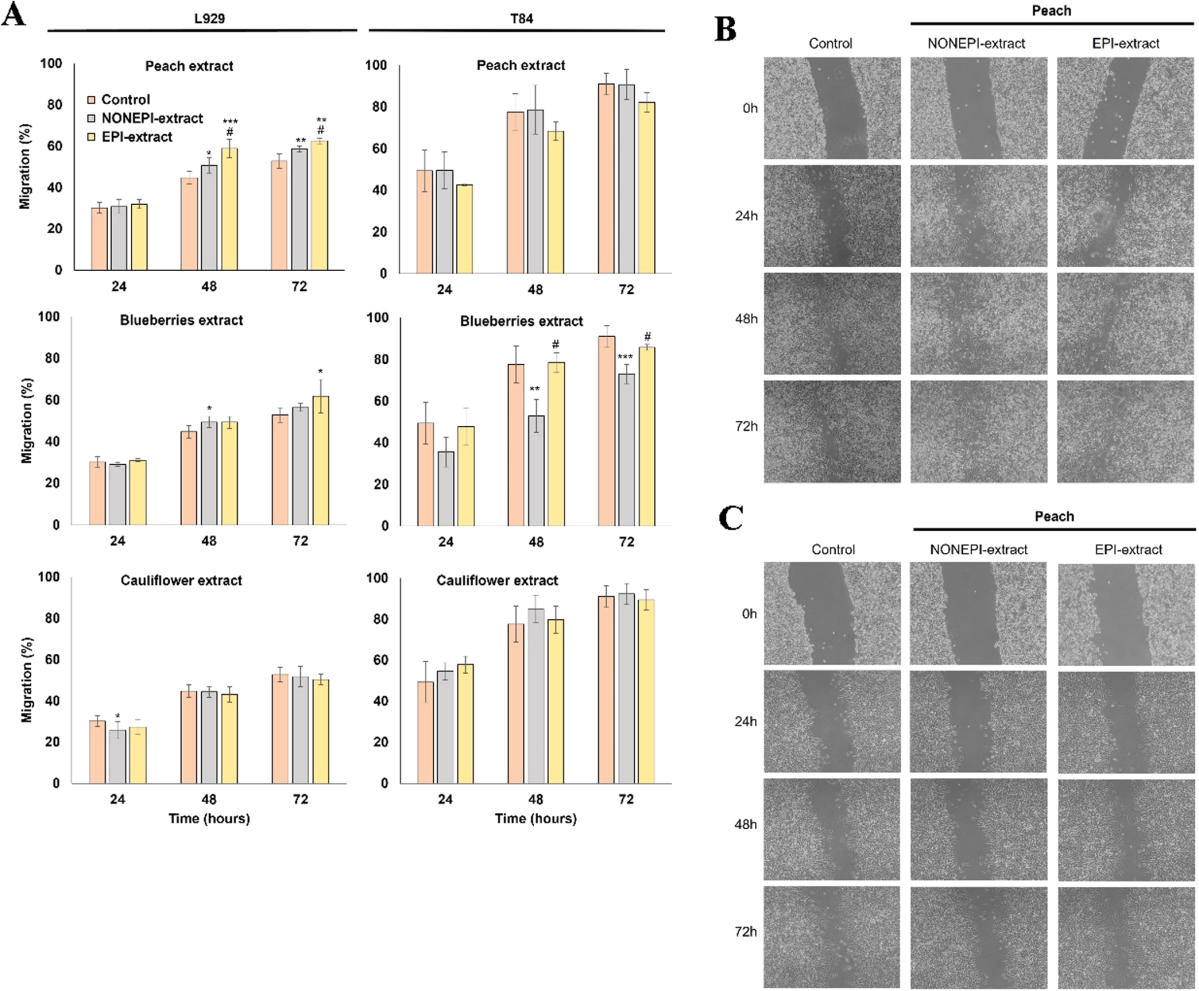
Effect of extracts on cell migration. (A) Quantitative analysis of cell migration in L929 and T84. Data are expressed as the mean ± standard deviation (SD) of at least three independent experiments performed in triplicate. *p* < 0.05; *p < 0.01; **p < 0.001 versus control cells (untreated); #*p* < 0.05 versus the NONEPI-extract. (B) Representative images of L929 cells treated with the Peach-EPI-extract and control (untreated) cells at different time points. (C) Representative images of T84 fibroblasts treated with both peach-EPI and -NONEPI-extract at the indicated time points.

### 3.6 Antioxidant Capacity of the Extracts

HT29 cells were employed to assess the *in vitro* antioxidant activity of the different extracts by determining their ability to protect cells against hydrogen peroxide-induced oxidative stress. Cells were pretreated with nontoxic concentrations of each extract for 24 h prior to exposure. As shown in Figure 8, the most pronounced protective effect was observed for the cauliflower-EPI-extract, which increased cell viability by 11.8% compared with the control group (*p* < 0.001) while cauliflower-NONEPI-extract did not significantly affect cell survival. In contrast, only the blueberry-NONEPI-extract also enhanced cell viability by 11.4% (*p* < 0.001), whereas the corresponding EPI-extract showed no significant effect. No significant difference effect was observed between both peach-EPI and peach-NONEPI-extracts.

**Figure 8:**
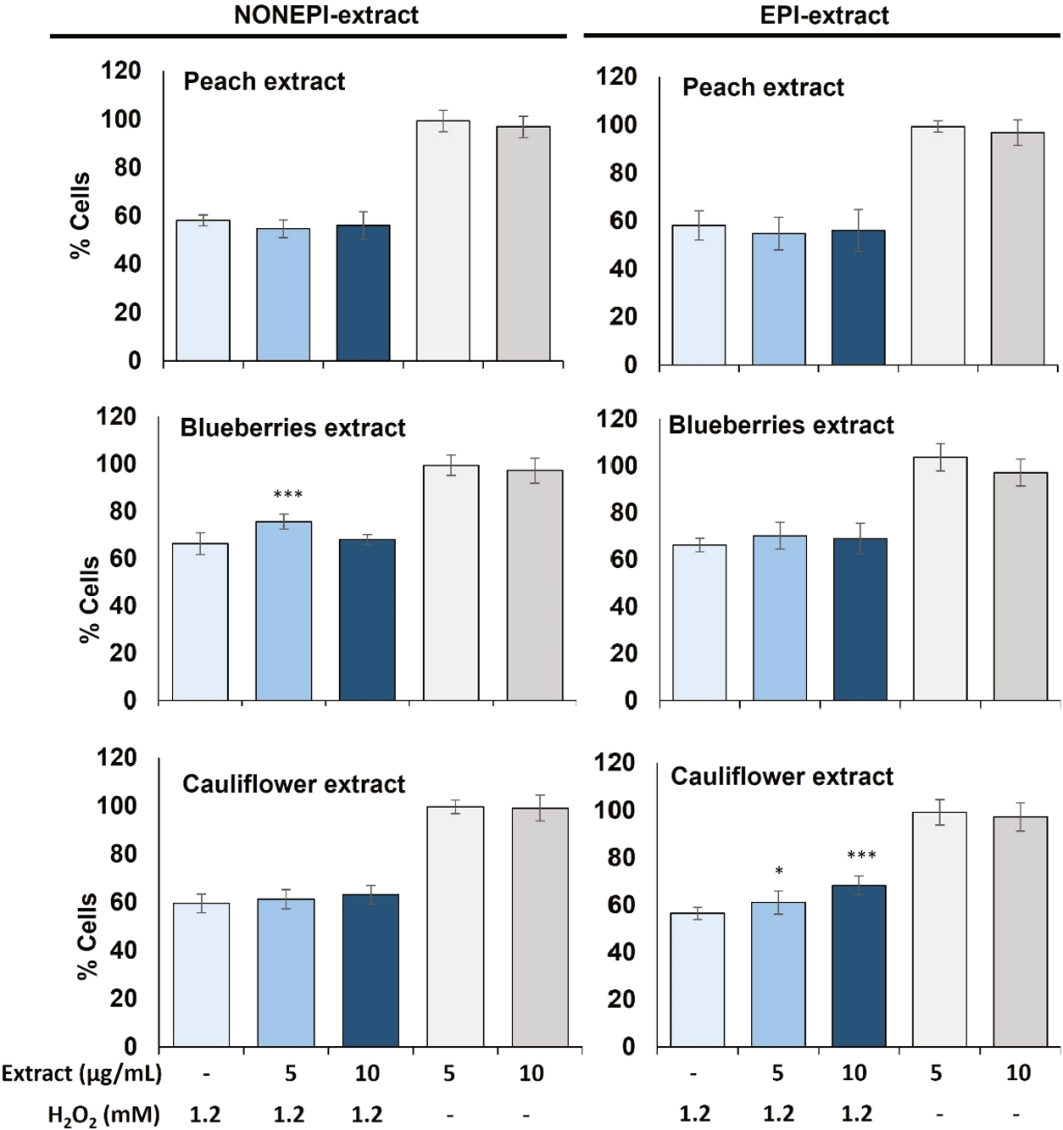
In vitro antioxidant activity of different extracts in HT29 cells. HT29 cell cultures were pretreated with nontoxic concentrations (5 and 10 μg/mL) of each extract for 24 h and subsequently exposed to hydrogen peroxide (H_2_O_2_). Data are presented as the mean ± standard deviation (SD) of eight replicates. *p* < 0.05; *p < 0.01; **p < 0.001 compared with H_2_O_2_-treated cells (control).

### 3.7 Analysis of the Ability of Extracts to Induce Detoxifying Enzymes

To evaluate the potential of the different extracts to enhance the activity of detoxifying enzymes GST and QR assays were performed. As shown in Figure 9A and Table 2, the most notable result was the increase in GST activity induced by the cauliflower-EPI-extract, which was 1,04-fold higher than that observed with the cauliflower-NONEPI-extract. Moreover, this extract exhibited greater GST activity than the untreated control (Figure 9). Similarly, the blueberry-EPI and blueberry-NONEPI-extracts also enhanced GST activity, showing comparable levels between them. In contrast, no significant changes in QR activity were detected between EPI and NONEPI-extracts in any of the tested samples, although in general they show a reduction in the expression of this enzyme when compared to the untreated control (Table 2 and Figure 9B).

**Figure 9:**
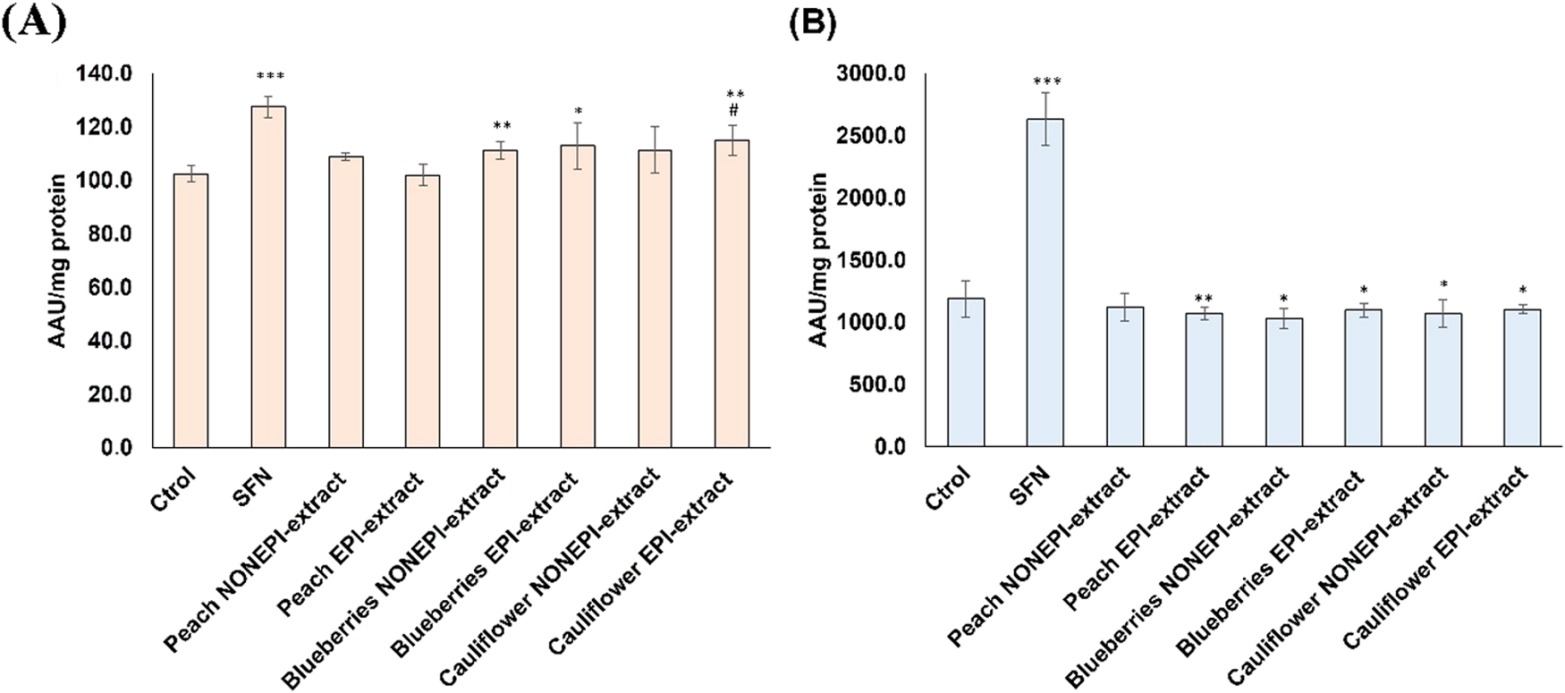
Graphical representation of effect of different extracts on the induction of detoxifying enzymes. Sulforaphane was used as a positive control. Enzyme activity is expressed as arbitrary activity units (AAU) per milligram of protein in the cytosolic fraction for (A) glutathione S-transferase (GST) and (B) quinone reductase (QR). Data are presented as the mean ± standard deviation (SD) of three independent experiments. *p* < 0.05; *p < 0.01; **p < 0.001 versus control cells (untreated); #*p* < 0.05 versus the respective NONEPI-extract.

**Table 2:** Induction of GST and QR Enzyme Activities after 48 h of Treatment with Different Extracts in HT29 Cells^a^

	GST		QR	
	AAU/mg protein	Induction Rate	AAU/mg protein	Induction Rate
Control	102.6 ± 3.03	1.00 ± 0.00	1187.5 ± 144.32	1.00 ± 0.00
Sulforaphane	127.5 ± 3.92***	1.12 ± 0.14	2631.4 ± 211.80***	2.24 ± 0.15
Peach NONEPI-extract	108.8 ± 1.49	1.06 ± 0.01	1123.8 ± 113.61	1.03 ± 0.06
Peach EPI-extract	102.0 ± 4.00	0.99 ± 0.04	1070.4 ± 48.35**	0.96 ± 0.06
Blueberries NONEPI-extract	111.3 ± 3.24**	1.08 ± 0.03	1030.0 ± 79.98*	0.95 ± 0.05
Blueberries *EPI-extract*	113.0 ± 8.73*	1.10 ± 0.08	1098.7 ± 55.77*	1.02 ± 0.07
Cauliflower NONEPI-extract	111.3 ± 8.72	1.08 ± 0.08	1068.9 ± 110.03*	0.97 ± 0.11
Cauliflower EPI-extract	115.1 ± 5.64^**#^	1.12 ± 0.05	1103.8 ± 33.15*	1.02 ± 0.07

a Sulforaphane was used as a positive control. Data are expressed as the mean ± standard deviation (SD). *p* < 0.05; *p < 0.01; **p < 0.001 versus control cells (untreated); #*p* < 0.05 versus the respective NONEPI-extract

### 3.8 Angiogenesis Test Using CAM Assay

The angiogenic or antiangiogenic potential of the different extracts was evaluated using the CAM assay, which assesses changes in vascular density and vessel length. As shown in Figure 10, the blueberry-EPI-extract was particularly noteworthy, as it significantly reduced vascular formation to 0.7-fold compared with the control (untreated) (*p* < 0.01). Moreover, this antiangiogenic effect was significantly stronger than that produced by the blueberry-NONEPI-extract (*p* < 0.01). In contrast, neither the peach or the remaining blueberry extracts (EPI and NONEPI) exhibited significant differences among themselves or relative to the PBS-treated control.

**Figure 10:**
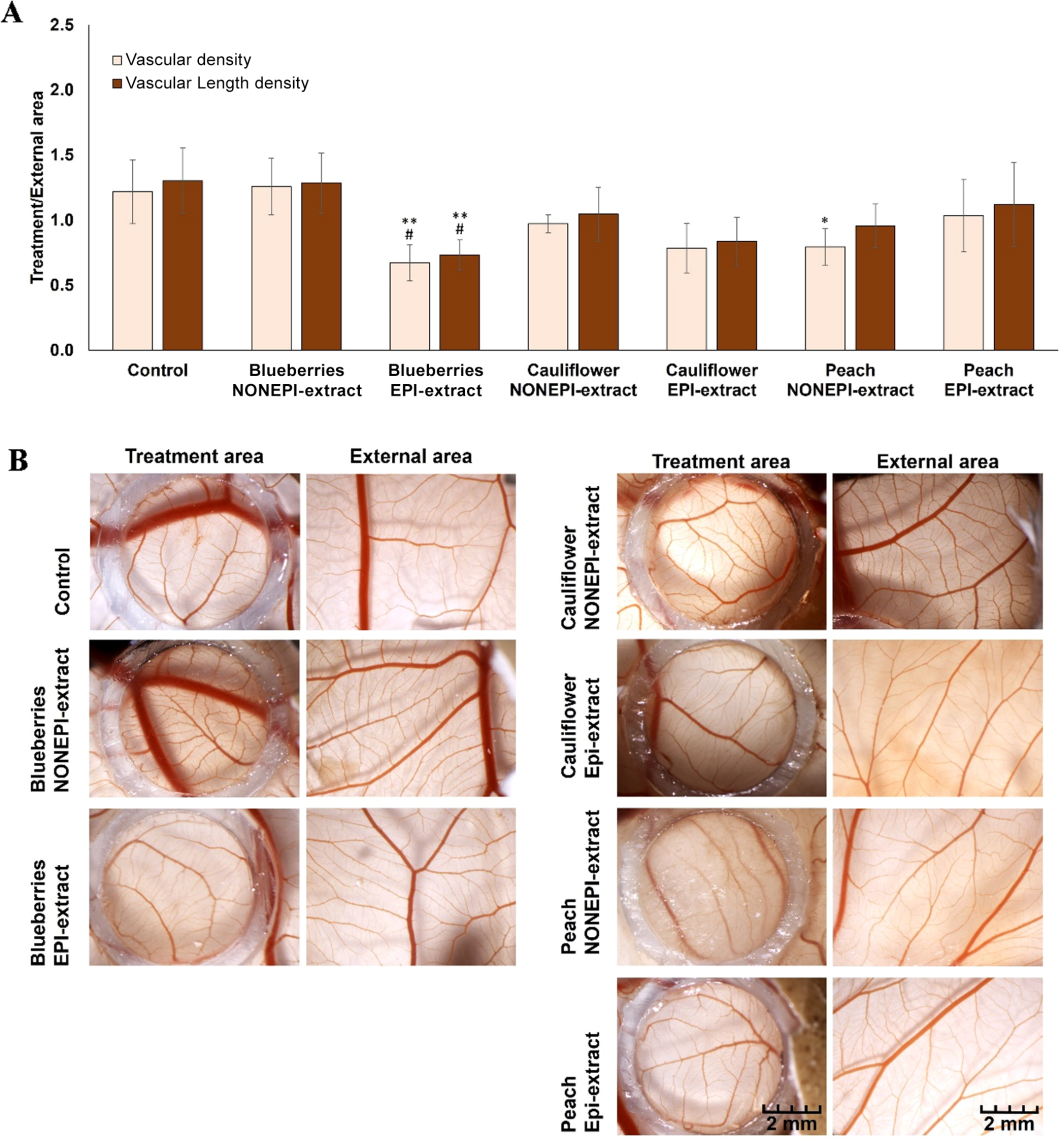
Testing for angiogenesis using the CAM assay. EPI and NONEPI-extract from blueberries, cauliflower, and peach were applied at a concentration of 100 μg/mL for 72 h of treatment. PBS-treated eggs were used as negative control. (A) Graphical representation of the vascular density and vascular length density measurements given by the “Vessel Analysis” plugin from FIJI software. (B) Images taken by Motic SMZ-171 stereo microscope of either the treatment area inside the ring and the outside of the ring. The scale bar shown in the figures equals to 2 mm. Results are given as mean ± SD of at least three eggs per experimental group. *: *p* < 0.05 and **: *p* < 0.01 compared to control eggs; #: *p* < 0.05 compared to the respective NONEPI-extracts.

## 4 Discussion

The present study combines ^1^H NMR-based metabolomics with functional biological assays to explore how different agricultural treatments modulate both the biochemical composition and the biological activity of fruit and vegetable extracts. While direct causal relationships cannot be established, this combined approach revealed consistent, matrix-dependent associations between treatment-related metabolic modulation and biological responses, including cell migration, antioxidant protection, detoxifying enzyme activity and angiogenesis. Although both metabolomic and biological data sets were analyzed, their integration is approached from a comparative perspective, providing insight into treatment-associated trends across matrices.

In addition, pathway analysis provided a complementary overview of the metabolic alterations, highlighting the involvement of central pathways related to carbohydrate metabolism, amino acid metabolism, and energy-related processes. Specifically, carbohydrate-related pathways such as starch and sucrose metabolism and galactose metabolism were predominantly affected in blueberry and peach, whereas amino acid metabolism, including alanine, aspartate and glutamate metabolism and arginine biosynthesis, together with TCA cycle-related pathways, showed a more pronounced contribution in cauliflower. These observations are consistent with the variations in sugars, amino acids, and organic acids identified by NMR, supporting the interpretation that agricultural practices are associated with coordinated changes in primary metabolism across matrices.

Berries belonging to the genus *Vaccinium*, including European blueberry (Vaccinium myrtillus) and North American blueberry (*Vaccinium corymbosum*), have received increasing scientific attention due to their exceptionally high content of flavonoids, anthocyanins, phenolic acids and tannins, which have been associated with a wide range of biological activities.^28^ Accordingly, blueberries are widely recognized for their high antioxidant capacity and health-promoting properties, largely attributed to these phenolic compounds. In particular, anthocyanins, phenolic acids and other flavonoids have been reported to reduce oxidative stress and modulate key cellular enzymes involved in detoxification and redox balance.^29^ In the present study, metabolic differences between treatments were mainly associated with sugars and amino acids: EPI extracts showed higher levels of glucose, fructose, asparagine and arginine, whereas NONEPI extracts contained higher levels of sucrose, threonine, alanine, valine, isoleucine and leucine. These shifts involved primary metabolites and did not include phenolic or flavonoid compounds typically linked to blueberry bioactivity, reflecting their lower abundance and limited visibility in ^1^H NMR profiles under the extraction conditions employed. Regarding the biological outcomes, the blueberry-NONEPI extract significantly reduced T84 cell migration while enhancing cellular viability under oxidative stress, suggesting potential antiproliferative and cytoprotective effects in this treatment group. Notably, only the blueberry-EPI extract exhibited a pronounced antiangiogenic effect in the CAM assay, consistent with previously reported vascular effects of blueberry phenolics. The absence of a clear correlation between this angiogenic response and the NMR-detected metabolite profiles suggests that compounds present below the NMR detection threshold, such as anthocyanins or proanthocyanidins, may contribute to this effect. This observation underscores the complexity of linking metabolic profiles with functional outcomes when using NMR-detected metabolites alone.

Cauliflower and other *Brassica* vegetables are increasingly recognized as valuable sources of bioactive compounds with potential health benefits. Previous studies have highlighted the presence of diverse classes of metabolites in cauliflower matrices, including amino acids, organic acids, polyols, phenolic compounds and glucosinolates.^30^ The metabolic composition of *Brassica* crops is known to be strongly influenced by agronomic practices, together with climatic conditions, genotype and nutrient availability. In particular, soil nitrogen supply has been reported as a key factor modulating both primary and secondary metabolism, with effects on amino acids, organic acids and nitrogen-containing secondary metabolites. Accordingly, comparisons between different cultivation systems have suggested that changes in fertilization and plant stress responses may significantly alter metabolite accumulation.^31^ In this context, cauliflower exhibited the most pronounced treatment-associated metabolic modulation among the matrices analyzed in this study. Cauliflower-EPI samples showed higher levels of multiple amino acids (leucine, isoleucine, alanine, valine, threonine, arginine, glutamate, glutamine, aspartate, asparagine, proline, tyrosine, histidine, tryptophan and phenylalanine), citrate, dimethylglycine, choline and uridine, whereas NONEPI samples showed increased malate and succinate. Together, these metabolic differences suggest broad modifications in nitrogen-related pathways and TCA cycle intermediates, consistent with a more extensive chemical response to treatment than that observed in blueberry and peach. These metabolomic findings are consistent with the biological results. Unlike blueberry, cauliflower extracts did not significantly alter cell migration in either L929 or T84 cells, suggesting that treatment-associated metabolic shifts were not translated into motility changes in these models. However, the cauliflower-EPI extract exerted the strongest protective effect against oxidative stress, increasing cell viability by 11.8% relative to the control, whereas the NONEPI extract had no significant impact. In addition, GST activity was markedly enhanced in the EPI extract, exceeding both NONEPI and control values, indicating that treatment-related metabolic changes may contribute to an enhanced detoxifying enzyme activity profile in cauliflower. Overall, the combined data indicates that cauliflower responded strongly at the metabolic level, particularly in amino acid pathway, and that these changes were accompanied by biological effects consistent with enhanced cytoprotection and detoxifying enzyme induction in the EPI condition. In contrast to blueberry, no angiogenic or cell migration effects were detected, reflecting matrix-specific differences in both chemistry and functional responses.

Regarding the third matrix analyzed in this study, peach fruits have been widely studied for their nutritional and functional properties, which are partly attributed to the presence of phenolic compounds such as chlorogenic acid, *p*-coumaric acid and flavonoid derivatives, particularly in the fruit peel. These phytochemicals have been associated with antioxidant and enzyme-modulating activities, including α-amylase inhibition, highlighting their potential contribution to health-related effects.^32^ However, the biological activity of peach extracts is not exclusively driven by phenolic compounds, since other primary metabolites may also play relevant roles depending on the biological assay and the extraction conditions employed. In this context, ^1^H NMR-based metabolomics provides a comprehensive overview of the primary metabolic composition of peach extracts, enabling the evaluation of treatment-associated changes in sugars, amino acids and organic acids that may contribute functional responses beyond those directly attributable to phenolic constituents. In this study, peach extracts displayed an intermediate treatment-associated metabolic response compared with blueberry and cauliflower. From a metabolomic perspective, peach-EPI samples showed higher levels of branched-chain amino acids (valine and isoleucine), glutamate, glutamine, malate, myo-inositol, sugars (glucose, fructose and galactose), shikimate, uridine, ethanol and one unidentified aromatic resonance tentatively associated with a nucleoside/nucleotide-related compound. In contrast, NONEPI samples were characterized by higher levels of aspartate, sucrose and phenylalanine. Regarding the biological assays, the peach-EPI extract significantly enhanced fibroblast migration in the wound-healing assay, with a stronger and more persistent effect than the NONEPI extract at both 48 and 72 h. The increase in metabolites such as myo-inositol, sugars and amino acids in EPI samples may help to contextualize the biological assay results observed in this study. Myo-inositol is an essential component for cell growth and membrane biosynthesis, acting as a precursor of phospholipids and signaling molecules,^33,34^ while branched-chain amino acids are known to promote anabolic pathways and wound-healing processes.^35^ In addition, soluble sugars provide the primary carbon substrates required for cellular metabolism.^36^ Unlike blueberries, peach-EPI and peach-NONEPI extracts did not produce significant effects on either angiogenesis or antioxidant activity, regardless of treatment, indicating that the NMR-detected metabolic changes were not functionally translated.

Taken together, these results suggest that regenerative agricultural practices can modulate plant metabolism in a manner that may be reflected in biologically relevant functional outcomes, although the nature of these effects is strongly dependent on the plant matrix. The combined metabolomic–biological approach adopted here provides a useful framework for the comparative evaluation of agricultural practices in relation to the chemical and functional properties of plant-derived foods. Importantly, these findings should not be interpreted as uniform increases in bioactivity, but rather as matrix-specific metabolic responses that may differentially influence health-related end points. Further studies combining complementary analytical platforms such as LC-MS and correlation-based approaches would help to further elucidate the relationship between metabolite composition and biological activity.

## Supplementary Material



## Data Availability

The data sets generated during and/or analyzed during the current study are available from the corresponding author on reasonable request.

## Notes

Metabolite assignment table including ^1^H NMR chemical shifts, multiplicities, and J-coupling constants for peach, blueberry, and cauliflower extracts; PCA score plots; PLS-DA permutation tests; KEGG pathway analysis figures and summary tables for EPI and NONEPI samples (PDF)
