# Supplementary material for: Comparative NMR-Based Metabolomic and Functional Assessment of Fruit and Vegetable Extracts under Regenerative Agricultural Practices
Source: J Agric Food Chem. 2026 Jul 3;74(27):21641–56. doi: 10.1021/acs.jafc.6c01741 (PMC13383750; doi:10.1021/acs.jafc.6c01741)
Supplement: Supplementary file 1 [file jf6c01741_si_001.pdf]

Supporting Information

Comparative NMR-based metabolomic and functional assessment of fruit and vegetable extracts under regenerative agricultural practices

Ana Isabel Tristán,[1,#] Gloria Perazzoli,[2,3,4,#] Ana Cristina Abreu,[1] Ana del Mar Salmerón,[1] Silvia Fernández,[1] Francisco Javier del Águila,[5] Juan Enrique Gázquez,[5] Antonio Fernández,[5] Consolación Melguizo,[2,3,4] José Prados,[2,3,4,*] and Ignacio Fernández[1,*]

[1] Department of Chemistry and Physics, Research Centre CIAIMBITAL, University of Almería, Ctra. Sacramento, s/n, 04120, Almería (Spain)

[2] Institute of Biopathology and Regenerative Medicine (IBIMER), Center of Biomedical Research (CIBM), University of Granada, 18100 Granada, Spain

[3] Instituto de Investigación Biosanitaria de Granada, ibs.GRANADA, 18012 Granada, Spain

[4] Department of Anatomy and Embryology, Faculty of Medicine, University of Granada, 18071 Granada, Spain

[5] Viagro S.A., Ctra. La Cañada-Viator, 04120 Almería, Spain.

Contents

Table S1. Metabolites identified in peach, blueberry, and cauliflower extracts, including ^{1}H NMR chemical shifts, multiplicities, coupling constants, and matrix occurrence.

Figure S1. PCA score plots derived from bucketed ^{1}H NMR spectra of peach, blueberry, and cauliflower extracts.

Figure S2. Permutation tests (n = 200) validating PLS-DA models for peach, blueberry, and cauliflower samples under EPI and NONEPI conditions.

Figure S3. KEGG pathway analysis based on significantly altered metabolites between EPI and NONEPI samples.

Table S2. Summary of KEGG pathway analysis results, including pathway enrichment statistics and impact values.

Table S1. Metabolites identified in peach, blueberry and cauliflower. The table reports the ^{1}H NMR spectral information used for metabolite assignment, including chemical shifts (ppm), signal multiplicities and *J*-coupling constants (Hz), together with the presence of each metabolite in the corresponding matrix (indicated by "X").

	**Metabolites**	**Chemical shift (δ_H, ppm), multiplicity and *J* (Hz)**	**Peach**	**Blueberry**	**Cauliflower**
Amino acids					
1	Valine	1.01 (d, *J* = 6.96 Hz), 1.06 (d, *J* = 6.96 Hz)	X	X	X
2	Leucine	0.98 (d, *J* = 6.41 Hz), 0.99 (d, *J* = 6.41 Hz)	X	X	X
3	Isoleucine	0.96 (t, *J* = 7.33 Hz), 1.03 (d, *J* = 6.96 Hz)	X	X	X
4	Threonine	1.34 (d, *J* = 6.49 Hz)	X	X	X
5	Alanine	1.49 (d, *J* = 7.37 Hz)	X	X	X
6	Arginine	1.51 (m), 1.74 (m), 1.93 (m), 3.24 (m)		X	X
7	Lysine	1.67 (m), 1.74 (m), 1.90 (m), 3.01(m)			X
8	GABA	1.90 (q, *J* = 7.19 Hz), 2.31 (t, *J* = 7.20 Hz), 3.02 (m)		X	X
9	Glutamine	2.14 (m), 2.46 (m)	X	X	X
10	Glutamate	2.05 (m), 2.13 (m), 2.39 (m)	X	X	X
11	Aspartate	2.64 (dd, *J* = 17.35, 9.34 Hz), 2.81 (dd, *J* = 12.23, 5.24 Hz)	X	X	X
12	Asparagine	2.82 (dd, *J* = 17.05, 8.19 Hz), 2.95 (dd, *J* = 16.88, 4.01 Hz)	X	X	X
13	Proline	3.21, 3.50, 4.12 (dd, *J* = 5.66, 8.06 Hz)			X
14	Histidine	7.11 (s), 7.90 (s)		X	X
15	Tyrosine	6.85 (m), 7.19 (m)		X	X
16	Phenylalanine	7.34 (m), 7.40 (m)	X		X
17	Tryptophan	7.14 (m), 7.22 (m), 7.30 (s), 7.48 (m), 7.73 (m)		X	X
Organic acids					
18	3-hydroxybutyric acid	1.14 (d), 2.28 (m)	X		
19	Acetic acid	1.92 (s)	X	X	X
20	Succinic acid	2.44 (s)	X		X
21	Malic acid	2.40 (dd, *J* = 15.47, 9.12 Hz), 2.69 (dd, *J* = 15.64, 3.45 Hz), 4.28 (dd, *J* = 9.61, 3.25 Hz)	X	X	X

22	Citric acid	2.54 (d, *J* = 15.57 Hz), 2.71 (d, *J* = 15.57 Hz)	X	X	X
23	Fumaric acid	6.53 (s)	X	X	X
24	Formic acid	8.47 (s)	X	X	X
25	Quinic acid	1.87 (dd), 1.65 (m), 2.04 (m)	X	X	
26	Shikimic acid	2.20, 2.75, 3.71 (m), 4.02 (m), 4.42 (m), 6.73 (m)	X	X	
Carbohydrates					
27	Glucose	3.20 (dd, *J* = 9.36, 8.02 Hz), 3.36 (m), 3.39 (m), 3.44 (m), 3.47 (dd, *J* = 9.69, 3.68 Hz), 3.70 (m), 3.81 (m), 3.88 (dd, *J* = 12.70, 2.01 Hz), 4.59 (d, *J* = 7.89 Hz), 5.19 (d, *J* = 3.77 Hz)	X	X	X
28	Fructose	3.53 (m), 3.69 (m), 3.80 (m), 3.86 (dd, *J* = 9.91, 3.40 Hz), 3.95 (m), 4.03 (dd, *J* = 12.55, 1.42 Hz), 4.08 (m)	X	X	X
29	Sucrose	3.48 (m), 3.65 (m), 3.80 (m), 4.18 (d, *J* = 8.70 Hz), 5.41 (d, *J* = 3.77 Hz)	X	X	X
30	Galactose	4.52 (d), 5.36 (d)	X		
31	Unidentified carbohydrate-related compound	3.40-3.90, 5.13 (d)			X
Phenolic compounds					
32	Chlorogenic acid	6.33 (d, J = 15.8 Hz), 7.58 (d, J = 15.8 Hz), 6.86 (d, J = 8.4 Hz), 7.04 (dd, J = 8.4, 2.1 Hz), 7.12 (d, J = 2.1 Hz), 1.96 (m), 2.03 (m)		X	
33	Unidentified flavonoid-type phenolic compound (1)	6.32 (d, J =1.8 Hz), 6.55 (d, J =1.8 Hz), 6.98 (d, J =8.7 Hz), 7.58 (dd, J = 8.7, 2.0 Hz), 7.73 (d, J = 2.0 Hz)		X	
34	Unidentified flavonoid-type phenolic compound (2)	6.36 (d), 6.55 (d)		X	
35	Epicatechin	7.11 (d, J = 1.7 Hz), 6.90 (m), 6.02 (d, J = 2.0 Hz), 6.04 (d, J = 2.0 Hz)		X	
36	Unidentified phenolic-like compound	6.95 (s), 7.69 (s)	X		

Others metabolites					
37	Ethanol	1.19 (t)	X		
38	Dimethylglycine	2.83 (s)			X
39	Ethanolamine	3.12 (m)			X
40	Choline	3.22 (s)	X	X	X
41	Myo-inositol	3.21 (t)	X	X	
42	Uridine	5.86 (d, J = 8.10 Hz), 5.90 (d, J = 4.72 Hz), 7.93 (d, J = 8.10 Hz)	X		X
43	Trigonelline	4.46 (s), 8.11 (m), 8.87 (m), 9.15 (s)	X		X
44	Fatty acids	0.87 (–CH_3, except n-3), 1.28 (–$(CH_2)_n$–), 1.57 (–CH_2–CH_2–COOR), 2.04 (–CH_2–CH=CH–,UFA), 2.35 (–CH_2–COOR), 5.32 (–CH=CH–, UFA)	X	X	X
45	Nucleoside/nucleotide-related compound	8.0 - 8.5 (s)	X		X

^{1}H NMR signal multiplicities are reported as follows: s, singlet; d, doublet; t, triplet; dd, doublet of doublets; m, multiplet.

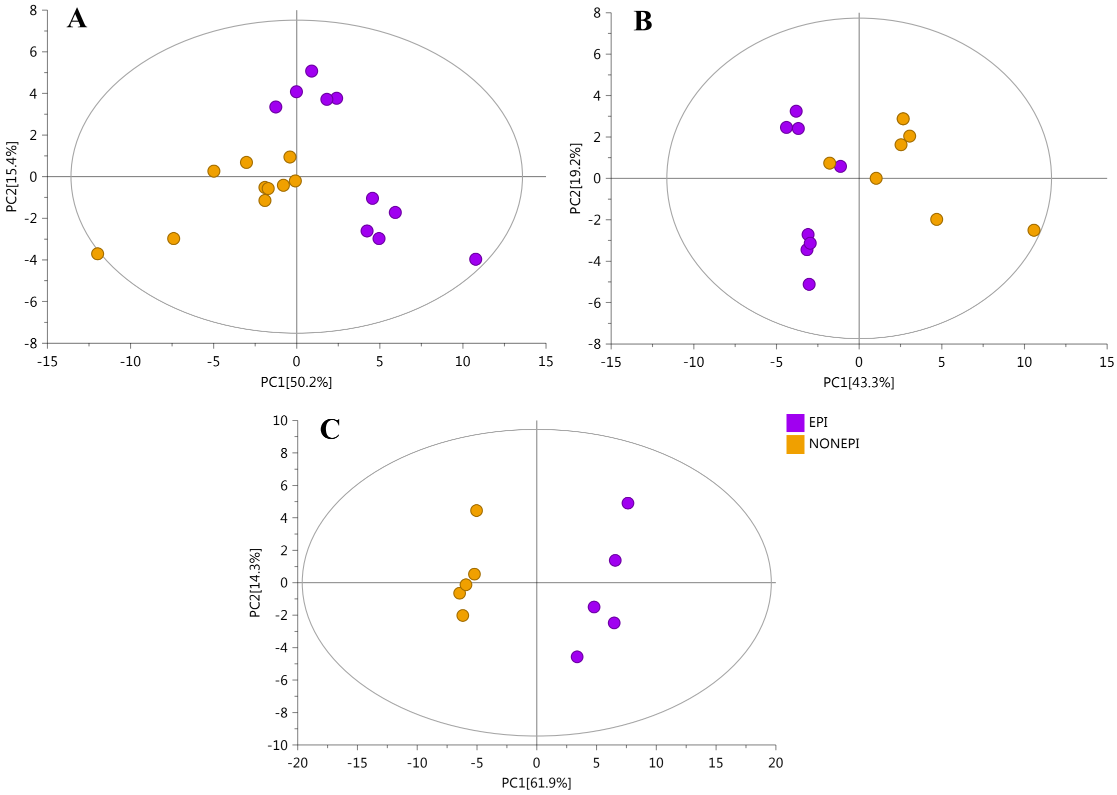

Figure S1. PCA score plots derived from bucketed ^{1}H NMR spectra of (A) peach, (B) blueberry and (C) cauliflower extracts.

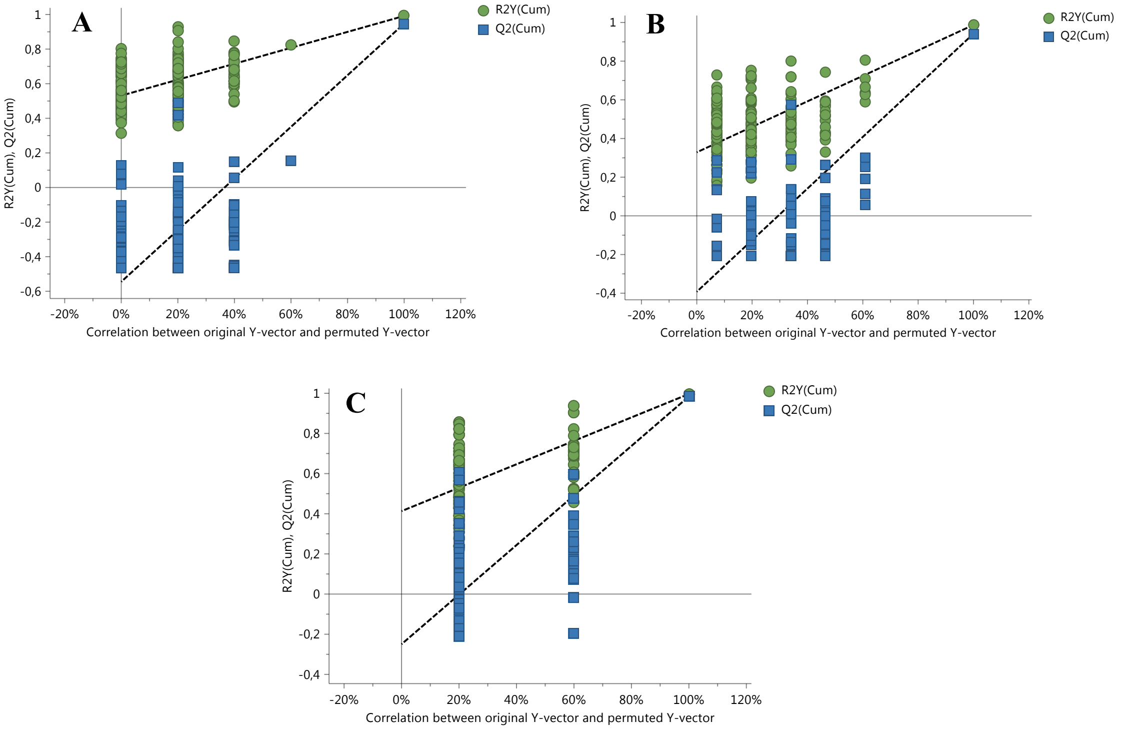

Figure S2. Permutation tests (n = 200) for PLS-DA models of (A) peach, (B) blueberry, and (C) cauliflower samples under EPI and NONEPI conditions. The original model R^2 and Q^2 values are higher than those of permuted models, with negative Q^2 intercepts, supporting model validity and excluding overfitting.

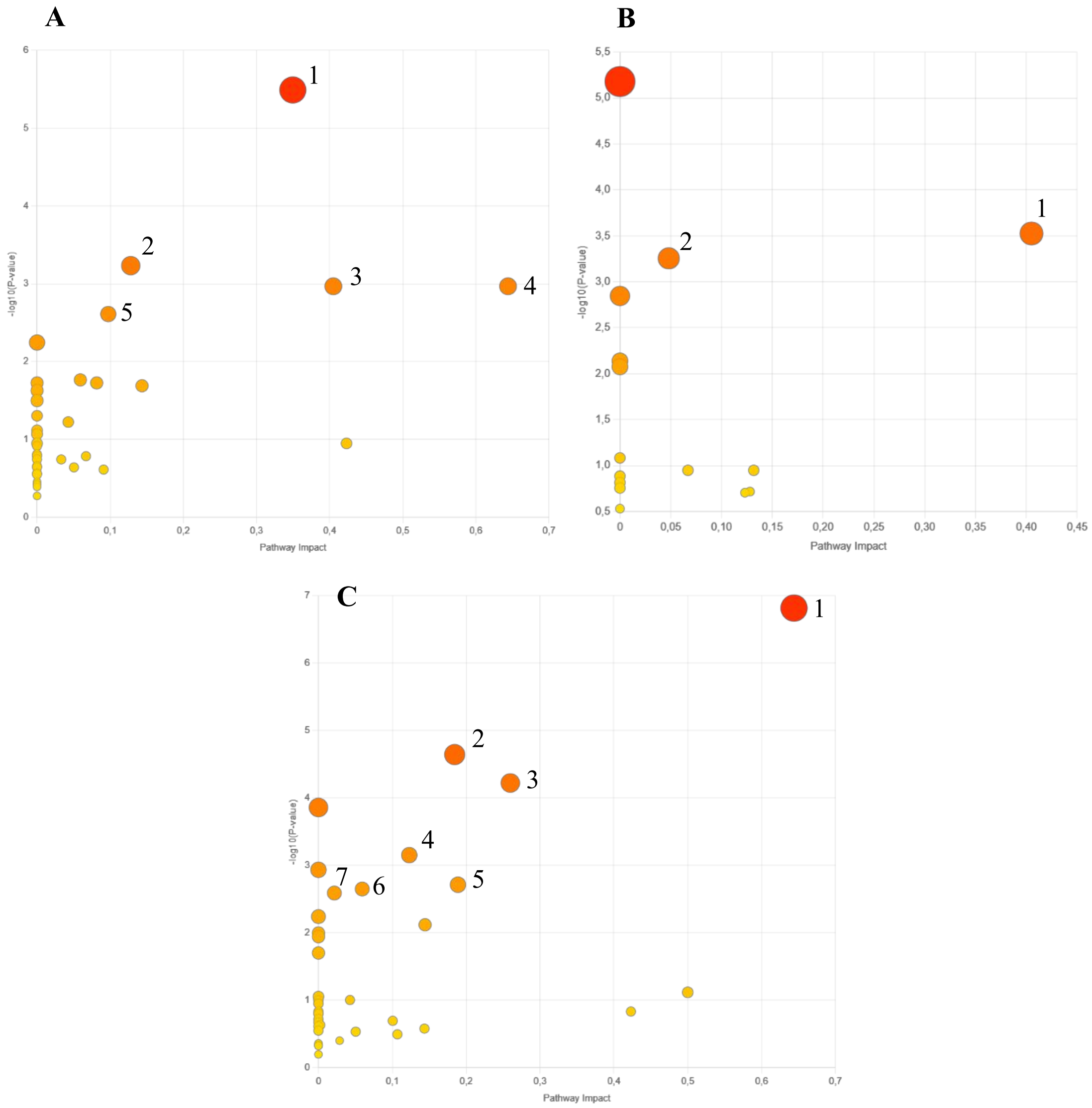

Figure S3. KEGG pathway analysis based on significantly altered metabolites between EPI and NONEPI samples for (**A**) peach, (**B**) blueberry, and (**C**) cauliflower. Pathways are ranked according to enrichment significance (−log(*p*)) and pathway impact values derived from topology analysis. Numerical labels correspond to the pathways listed in Table S2. Only the most relevant pathways are shown.

Table S2. Summary of KEGG pathway analysis results based on significantly altered metabolites between EPI and NONEPI samples. The table includes pathway name, number of matching metabolites, *p*-values, −log(*p*), Holm-adjusted *p*-values, false discovery rate (FDR), and pathway impact values.

	Pathway name	Matching metabolites	*p*	-log (*p*)	*p* (Holm)	*p* (FDR)	Impact
Peach							
1	Galactose metabolism	5/27	$3.22\cdot10^{-6}$	5.49	$2.97\cdot10^{-4}$	$2.97\cdot10^{-4}$	0.35
2	Arginine biosynthesis	3/18	$5.85\cdot10^{-4}$	3.23	0.05	0.02	0.13
3	Starch and sucrose metabolism	3/22	$1.08\cdot10^{-3}$	2.97	0.10	0.02	0.41
4	Alanine, aspartate and glutamate metabolism	3/22	$1.08\cdot10^{-3}$	2.97	0.10	0.02	0.64
5	Glyoxylate and dicarboxylate metabolism	3/29	$2.45\cdot10^{-3}$	2.61	0.22	0.04	0.10
Blueberry							
	**Pathway name**	**Matching metabolites**	***p***	**-log (*p*)**	***p* (Holm)**	***p* (FDR)**	**Impact**
1	Starch and sucrose metabolism	3/22	2.98·10-4	3.53	0.03	0.01	0.41
2	Galactose metabolism	3/27	5.56·10-4	3.26	0.05	0.02	0.05
Cauliflower							
	**Pathway name**	**Matching metabolites**	***p***	**-log (*p*)**	***p* (Holm)**	***p* (FDR)**	**Impact**
1	Alanine, aspartate and glutamate metabolism	6/22	$1.52\cdot10^{-7}$	6.82	$1.40\cdot10^{-5}$	$1.40\cdot10^{-5}$	0.64
2	Glyoxylate and dicarboxylate metabolism	5/29	$2.27\cdot10^{-5}$	4.64	$2.06\cdot10^{-3}$	$1.04\cdot10^{-3}$	0.18
3	Arginine biosynthesis	4/18	$5.99\cdot10^{-5}$	4.22	$5.39\cdot10^{-3}$	$1.84\cdot10^{-3}$	0.26
4	Glycine, serine and threonine metabolism	4/33	$7.05\cdot10^{-4}$	3.15	0.06	0.01	0.12
5	Citrate cycle (TCA cycle)	3/20	$1.94\cdot10^{-3}$	2.71	0.17	0.02	0.19
6	Carbon fixation by Calvin cycle	3/21	$2.24\cdot10^{-3}$	2.65	0.19	0.03	0.06
7	Phenylalanine, tyrosine and tryptophan biosynthesis	3/22	$2.57\cdot10^{-3}$	2.59	0.22	0.03	0.02
